# Are social isolation, lack of social support or loneliness risk factors for cardiovascular disease in Australia and New Zealand? A systematic review and meta‐analysis

**DOI:** 10.1002/hpja.592

**Published:** 2022-03-31

**Authors:** Rosanne Freak‐Poli, Aung Zaw Zaw Phyo, Jessie Hu, S. Fiona Barker

**Affiliations:** ^1^ Department of Epidemiology and Preventive Medicine School of Public Health & Preventive Medicine Monash University Melbourne Australia

**Keywords:** cardiovascular diseases, interpersonal relations, loneliness, social isolation, social support

## Abstract

**Background:**

An international systematic review concluded that individuals with poor social health (social isolation, lack of social support or loneliness) are 30% more likely to develop coronary heart disease (CHD) and stroke. Notably, the two included Australian papers reported no association between social health and CHD or stroke.

**Objective:**

We undertook a systematic review and meta‐analysis to investigate the association between social isolation, lack of social support and loneliness and cardiovascular disease (CVD) incidence among people living in Australia and New Zealand.

**Methods:**

Four electronic databases were systematically searched for longitudinal studies published until June 2020. Two reviewers undertook title/abstract screen and one reviewer undertook full‐text screen and data extraction. Quality was assessed using the Newcastle – Ottawa Quality Assessment Scale.

**Results:**

Of the 725 unique records retrieved, five papers met our inclusion criteria. These papers reported data from three Australian longitudinal datasets, with a total of 2137 CHD and 590 stroke events recorded over follow‐up periods ranging from 3 to 16 years. Reports of two CHD and two stroke outcomes were suitable for meta‐analysis. The included papers reported no association between social health and incidence of CVD in all fully adjusted models and most unadjusted models.

**Conclusions:**

Our systematic review is inconclusive as it identified only a few studies, which relied heavily on self‐reported CVD. Further studies using medical diagnosis of CVD, and assessing the potential influence of residential remoteness, are needed to better understand the relationship between social health and CVD incidence in Australia and New Zealand.

**Abbreviations**
CVD: cardiovascular disease
DSSI: Duke Social Support Index
MOSS: 19‐point Medical Outcomes Study Social Support Survey
SSI: Social Support Index

## 1 INTRODUCTION

In Australia, cardiovascular disease (CVD) dominates as the greatest cause of Australia's morbidity, mortality and healthcare expenditure.^1^ Similarly, in New Zealand CVD is a leading cause of premature mortality^2^ and monopolises nearly a quarter of health expenditure on noncommunicable diseases.^3^ In 2003, an Expert Working Group of the National Heart Foundation of Australia^4^ concluded from a systematic review that there is strong and consistent evidence of an independent causal association between social isolation and the causes and prognosis of coronary heart disease (CHD). Yet our understanding of the link between social health and CVD is limited, especially compared to other key CVD risk factors highlighted by the National Heart Foundation of Australia, including elevated cholesterol or blood pressure, diabetes, significant family history, smoking, poor nutrition, physical inactivity, adiposity and depression.^5^ Social health refers to an individual's ability to form satisfying and meaningful relationships, their ability to adapt in social situations, and their interactions with and support from other people, institutions and services.^6^ The concepts of social isolation, social support and loneliness are often discussed in relation to social health.^7^ Social isolation is *“an objective state in which a person has minimal contact with others and low involvement in local community life”*.^8^ Social support is a subjective measure of how social connections are operationalised, and loneliness “*is a subjective, unwelcome experience of lack of or loss of companionship”*.^8^ Assessment of social health varies and the inconsistency is a common limitation of this research area.^9^, ^10^ However, there is a helpful framework to compare and contrast common tools.^10^ Furthermore, a recent theoretical debate has emerged to separate the concepts of social isolation, social support and loneliness, as they likely have different implications for health and well‐being.^7^ Before the COVID‐19 outbreak, 50% of Australians reported feeling lonely at least 1 day a week, 25% reported currently experiencing an episode of loneliness, and 10% reported that they lack social support.^11^ Prevalence rates seemed to be slightly lower in New Zealand; 36% reported feeling lonely at least 1 day a week and 14% reported feeling lonely all, most or some of the time.^12^

A number of conceptual frameworks illustrate the pathways between social health and health,^13^, ^14^, ^15^, ^16^, ^17^, ^18^, ^19^, ^20^ with several^13^, ^18^, ^20^, ^21^ being particularly relevant to our review as they describe the link between poor social health and CVD. The main, broad pathway that tends to be described is from poor social health, through molecular mechanisms, health behaviours, and chronic disease risk‐factors, leading to chronic mental and physical ill‐health and mortality; with each step being impacted by socio‐demographics, the sociological environment, and personality.^18^, ^20^, ^22^, ^23^ For example, being socially isolated or feeling lonely can overstimulate the body's stress response through increased levels of the stress hormone cortisol, raise blood pressure and decrease blood flow to vital organs through higher tonic vascular resistance, impair the immune system's ability to fight infections through lower production of white blood cells, and reduce sleep quality leading to less restorative sleep and daytime fatigue.^24^ People with poor social health also tend to have more unhealthy lifestyles, such as undertaking less physical activity or eating unhealthily, which increases their risk of CVD.^25^ Additionally, a bi‐directional pathway is also described “*with health and social relationships interacting to influence each other, in virtuous circles or spirals of despair”*.^14^ The bi‐directional pathway also incorporates the health selection model, which explains how deterioration in health (such as a CVD event or decline in cognitive functioning) may limit or reduce social involvement, which leads to greater ill‐health. Hence, poor social health likely impacts health and well‐being over the life‐course.

In 2016, an international systematic review of 23 studies concluded that individuals with poor social health were 30% more likely to develop CHD and stroke.^26^ The systematic review included 181 006 participants, aged 18 years and over, mainly from Europe (38%) or North America (33%), followed from 1965 to 1996 for 3‐21 years. Notably, the systematic review only included two Australian papers, which reported that a combined measure of social isolation and social support was not associated with CHD or stroke in fully adjusted models.^27^, ^28^ No eligible studies were identified from New Zealand. Given the unique geographical spread of the Australian and New Zealand populations, along with differences in political and cultural support systems (especially in terms of social systems and health care), research undertaken in other countries may not be generalisable. With the rise of CVD in Australia and New Zealand, along with the emerging knowledge of the role of social health in CVD, it is important to further the understanding of these issues in order to better address and mitigate them.

The aim of this systematic review is to investigate the association of social isolation, lack of social support and loneliness with CVD incidence among people living in Australia and New Zealand.

## 2 METHODS

This systematic review and meta‐analysis was conducted in accordance with the Preferred Reporting Items for Systematic Reviews and Meta‐Analyses^29^ (PRISMA) statement. Our protocol was followed unless otherwise defined (Prospero CRD42018093503).

### 2.1 Criteria

We included longitudinal observational data. We included studies conducted in the general population (not clinical location or for specific health reasons), generalisable to people living in Australia and New Zealand of all ages. We included the social health measures of social isolation, social support and loneliness. The primary outcome was incidence of CVD, obtained through self‐report or medical records.

### 2.2 Search methods

We searched four databases from the earliest record to 21st June 2020 (Appendix A). There were no language or date restrictions. One author (AZZP) scanned the references of included studies, and several relevant review articles identified in the initial search, for additional studies and found one additional article. The first authors of each included paper were asked if they knew of any additional studies which might be relevant. No additional studies were supplied.^30^, ^31^, ^32^

### 2.3 Data collection and analysis

Papers were provided an ID based on the first author's last name and year of publication. Two people (authors or contributors Noria Akbari, Aghnia Naim) undertook an initial screening of titles and abstracts independently, with discrepancies included in the full‐text screen. There were no relevant non‐English articles or conference abstracts. One author (RF) screened all full‐text papers and performed data extraction. A second author (AZZP) checked the extracted data. Two authors (RF & AZZP) independently assessed the quality of included studies using the Newcastle – Ottawa Quality Assessment Scale,^33^ with discrepancies resolved with independent assessment by a third author (FB). A score of eight or nine was deemed as a low risk of bias. In our protocol we stated the use of the STROBE Statement,^34^ however it was developed as a guideline for reporting observational studies rather than assessing quality.

### 2.4 Data synthesis

Meta‐analysis followed the Cochrane Collaboration^35^ guidance, with at least two studies required. We converted odds ratios^36^ and hazard ratios^37^ to relative risks. We initially misunderstood the available methodology,^38^ and were unable to convert the findings from a continuous exposure to a categorical exposure (as recorded in our protocol). Statistical heterogeneity was evaluated by using the *I* ^2^ statistic^35^ and the metan STATA command for the 95% confidence intervals, and funnel plots and Egger's test were used to assess publication bias (added since protocol). We intended to report by CVD type, gender, and partner status, dichotomous baseline data collection year, dichotomous length of follow‐up and by country (Australia vs. New Zealand).

## 3 RESULTS

### 3.1 Description of studies

**FIGURE 1:**
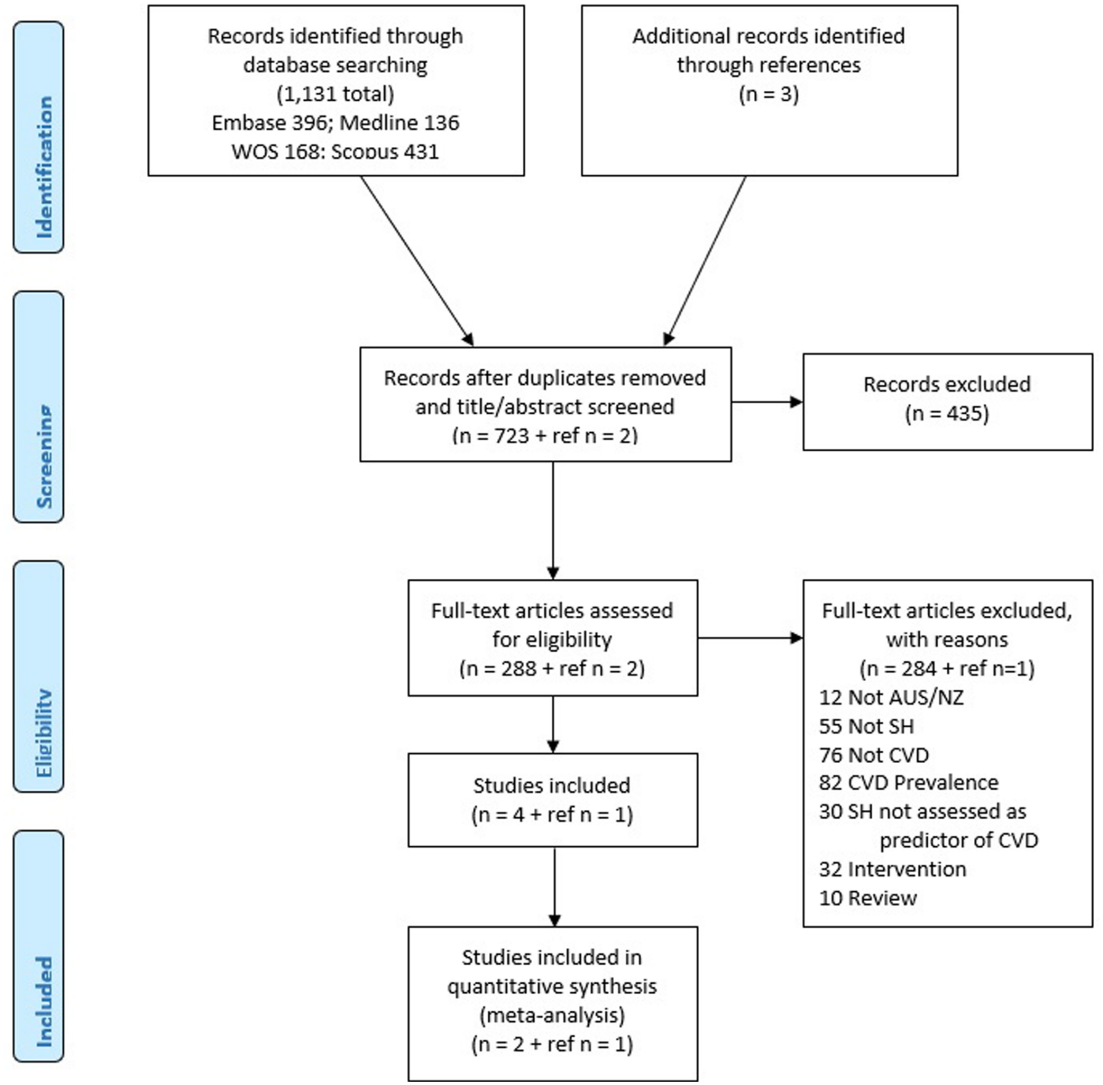
Flow diagram of review process

Five papers met our inclusion criteria (Figure 1, Table 1), which encompassed the two Australian studies included in the international systematic review.^26^ All samples were part of large Australian longitudinal cohort studies of non‐institutionalised adults. Three papers (Strodl 2003,^27^ Strodl 2008,^28^ Byles 2015^39^) used wave 2 data from the Australian Longitudinal Study on Women's Health (ALSWH) cohort born between 1921 and 1926 (aged 70 years or more at baseline). ALSWH was established in 1996 as a nationally representative cohort, with recruitment through Medicare records, people living in rural/remote double sampled and women too ill to participate excluded. Strodl 2003^27^ and Strodl 2008^27^, ^28^ further restricted the sample by excluding prevalent CHD or prevalent stroke at baseline (wave 2), while Byles 2015^39^ stratified by prevalent or incidence of stroke. The ALSWH collected the Duke Social Support Index (DSSI) which incorporates both aspects of social isolation and social support.

**TABLE 1:** Characteristics and relevant findings of the five included papers

ID	Sample	Eligibility	Demographics	Social health	Cardiovascular disease	Adjustment; Stratifications	Relevant findings	Limitations^a^
Strodl 2003^27^; ALSWH	1996 Baseline, 3 y follow‐up (1999); Australia; n = 6994 to 7759 depending on missing data	No prevalent CHD at baseline; did not die, withdraw or lost by wave 2 (1999) of ALSWH 1921‐1926 cohort recruited via Medicare database, with rural/remote double sampled, and excluded women too ill to participate	100% female; Age 70‐75 y; Education: 31% no formal, 16% tertiary; 40% major city [Calculated T1]	Duke Social Support Scale (DSSS), 11 items, categorised as low‐fair (<=26), high (27‐29) and very high (30‐33); via mail	Self‐reported symptomatic CHD through "*a doctor had told them in the previous 3 y that they had a diagnosis of heart disease such as angina or heart attack*", via mail; 3 y incidence	Partner status, time pressure, mental health index, perceived stress, remoteness, education; NR (female only)	Among 7759 women initially aged 70‐75, low‐fair DSSS scores predicted the new diagnosis of symptomatic CHD over the 3‐y period in univariable analyses (events = 489; compared to very high DSSS; low‐fair OR 1.41, 95% CI 1.11‐1.79 *P *<= .001; high OR 1.13, 95% CI 0.91‐1.39 *P* > .05) but not after adjusting for other psychosocial variables (results not reported)	Self‐reported CHD without clinical verification
Strodl 2008^28^; ALSWH	1996 Baseline, 3 y follow‐up (1999); Australia; n = 7839 to 9113 depending on missing data	No prevalent stroke at baseline; did not die, withdraw or lost by wave 2 (1999) of ALSWH 1921‐1926 cohort recruited via Medicare database, with rural/remote double sampled, and excluded women too ill to participate	100% female; Age 70‐75 y; Education: 32% no formal, 16% tertiary; 40% metropolitan [Calculated T1]	Duke Social Support Scale (DSSS), 11 items, categorised as low‐fair (<=26), high (27‐29) and very high (30‐33); via mail	Self‐reported stroke through "*if a doctor had told them in the previous 3 y that they had had a stroke*", via mail survey; 3 y incidence	None as univariable not statistically significant. If it were; hypertension, heart disease, diabetes, risk for malnutrition, obesity, activity level alcohol status; NR (female only)	Among 8907 women initially aged 70‐75, there was no association between DSSS categories and 3‐y incidence of stroke (events = 170; compared to very high DSSI; low‐fair OR 0.88, 95% CI 0.62‐1.25; high *incorrect in paper* OR 0.76, 95% CI 0.52‐1.12).	Self‐reported stroke without clinical verification; Survivor bias as survived to age 70‐75 and excluded stroke death during observation; residual confounding through unmeasured depression; 3 y follow‐up possibly underpowered
Byles 2015^39^; ALSWH	1996 Baseline, 3 y follow‐up (1999); Australia; n = 10 434	Did not die or withdraw by wave 2 (1999) of ALSWH 1921‐1926 cohort recruited via Medicare database, with rural/remote double sampled, and excluded women too ill to participate	100% female; Age 70‐75 y, 74.9 + 1.4SD; Education: 32.4% no formal, 3.8% university; 61.3% metropolitan [Calculated T2]	Duke Social Support Scale (DSSS), 11 items; via mail	Self‐reported stroke, via mail survey; baseline prevalent and 3 y incidence	NR; NR (female only)	[Calculated from T2] Among 10 434 women initially aged 70‐75, women without self‐reported stroke were more likely to have higher DSSS scores at baseline (n = 9738; DSSS 32.1 + 5.3SD) compared to women with prevalent stroke at baseline (n = 407; DSSI 30.8 + 5.5SD, *P* < .001) or incidence of stroke over 3 y (n = 289; DSSS 30.1 + 6.5SD, *P* < .001)	Self‐reported stroke without clinical verification; Survivor bias as survived to age 70‐75, those with a disability less likely to participate, excluded stroke death during observation
Simons 2013^40^; Dubbo	1988‐1989 Baseline, 8 y & 16 y follow‐up; Dubbo, New South Wales, Australia; n = 2805	Prior CHD or stroke permitted. From the Dubbo Study non‐institutionalised, not too ill or disabled to attend at initiation in 1988‐1989, born <1930, recruited through GPs and electoral records	Simons 2011: 56.0% female; Age >60 y, F: 69.6 + 7.3, M: 68.6 + 6.7 Simons 1991: Education: Primary F: 3%; M: 5%; Tertiary F: 1%; M: 4%; Socioeconomic status (0‐100) mean F: 33.4, M: 32.8	19‐point Medical Outcomes Study Social Support Survey. “Poor social support” categorised as expressed reservation about their social support situation (19.6%); via face‐to‐face interview	CHD and ischemic stroke incidence. Prevalence through self‐report or resting ECG. Incidence based on clinical diagnosis	(1) age, sex, and marital status (2) conventional predictors of CVD: smoking, alcohol, hypertension, diabetes, impaired peak expiratory flow, prior coronary heart disease, prior stroke, atrial fibrillation, physical disability, self‐rated health; NR	Among 2805 Australians aged 60+ followed for 16 y, social support (MOSS) did not precinct CHD events (CHD; 8 y HR 1.08, 95% CI 0.91‐1.29; 16 y events = 1088, HR 1.03, 95% CI 0.88‐1.20) or ischemic stroke (IS; 8 y HR 1.24, 95% CI 0.93‐1.65; 16 y events = 420, HR 1.20, 95% CI 0.88‐1.42) at 8 or 16 y after adjustment for age, sex, and marital status (model 1) or conventional predictors of CVD (model 2; CHD: 8 y HR 1.01, 95% CI 0.83‐1.21; 16 y HR 0.97, 95% CI 0.82‐1.14; IS 8 y HR 1.10, 95% CI 0.81‐1.50; 16 y HR 1.03, 95% CI 0.80‐1.33). Events not reported	Measurement of social support (eg subjective feelings vs. objective living arrangements) remains unclear; Healthy survivorship as 27% declined as “too old, too tired or too unwell”; Characteristics assumed to be stable during observation period
Sahle 2020^45^; HILDA	2003 Baseline, 10 y follow‐up (annually till 2013); Australia, nationally representative; n = 11 637	Without self‐reported non‐communicable disease (including heart disease, circulatory diseases), aged ≥21 y and have psychosocial measures from HILDA non‐institutionalised at initiation in 2001	51.9% female; Age ≥21 y, F: 44.3 + 15.9, M: 43.9 + 15.6; Education NR; SEIFA reported in deciles (~10% in each category)	The Social Support Index (SSI), (range −30, +30) sum of 10 statements, higher scores suggest lower loneliness; via mail	Self‐reported heart disease via mail survey; prevalent and 3 y incidence	(1) sociodemographic (age, marital status, education, Statistics Socio‐Economic Indexes), (2) 1 + lifestyle (smoking, alcohol, dietary pattern, physical activity), (3) 2 + BMI, high blood pressure, and (4) 3 + health‐related quality of life and co‐existing psychosocial factors; Stratified by gender	Among 11 637 adults aged >21 y, 3.6% of women (51.9% of sample) and 5.0% of men self‐reported heart disease over 10 y. Social support (SSI) was not associated with heart disease among women (events = 247; univariable OR 0.99, 95% CI 0.95, 1.03; adjusted OR 0.99, 95% CI 0.96, 1.03) or men (events = 313; univariable OR 0.98, 95% CI 0.96, 1.00; adjusted OR 0.99, 95% CI 0.98, 1.04) before or after adjustment for confounders	Self‐reported stroke without clinical verification; residual confounding through unmeasured variables; not generalisable to homeless or institutionalised

Abbreviations: ALSWH, Australian Longitudinal Study on Women's Health; CHD, coronary heart disease; Dubbo, The Dubbo Study; F, female; HILDA, household, income and labour dynamics in Australia; M, male; y, years.

^a^ Author identified (relevant) biases and limitations.

One included paper (Simons 2013^40^) used data from the Dubbo Study which was established in 1988 as a representative sample of community‐dwelling people born before 1930 (aged 60 years at baseline) living in Dubbo, New South Wales.^41^, ^42^, ^43^ As Simons 2013^40^ was a short report, the author recommended obtaining study details from prior publications.^41^, ^42^, ^43^, ^44^ Dubbo Study participants were recruited through general practitioners and electoral records, and excluded people too ill or disabled to attend data collection. Simons 2013^40^ did not put further restrictions on the sample, and included people with prevalent CHD and stroke at baseline. The Dubbo Study collected the 19‐point Medical Outcomes Study Social Support Survey (MOSSS) which assesses social support.

One included paper (Sahle 2020^45^) used data from the Household, Income and Labour Dynamics in Australia (HILDA) cohort which was established in 2001 as a nationally representative sample of households occupying private dwellings. HILDA households were recruited using census data and participants needed to be aged 21 years or older. Sahle 2020^45^ further excluded prevalent self‐reported non‐communicable disease (including heart disease and circulatory diseases) at baseline (2003 survey). HILDA collected the Social Support Index (SSI) which assesses loneliness.

### 3.2 Risk of bias

All studies were rated as high risk of bias, with NOS scores ranging between four and seven stars (Appendix C). All studies lost a point in the “*Comparability*” category for not adjusting for our list of most important factors. Most studies lost a point in the “*Outcome*” category as assessment of CVD was self‐report and the follow‐up was not long enough (defined as 5‐year or more).

### 3.3 Findings

The five included papers reported six CVD outcomes; two studies assessed CHD (Strodl 2003^27^ and Simons 2013^40^), three studies assessed stroke (Strodl 2008,^28^ Byles 2015^39^ and Simons 2013^40^), and one study assessed heart disease (Sahle 2020^45^). Social health was not associated with incidence of CVD in fully adjusted models and most unadjusted models.

### 3.4 Meta‐analysis

Methodological considerations were required prior to meta‐analysis. Strodl 2008^28^ and Byles 2015^39^ used the same source data, follow‐up period, social health exposure and CVD outcome. We chose Strodl 2008^28^ (rather than Byles 2015^39^) in the meta‐analysis as their analysis considered the social health exposure as a potential predictor of the incidence of CVD outcome. The four remaining included papers chose to assess the exposure as either categorical (n = 3; Strodl 2003,^27^ Strodl 2008,^28^Simons 2013^40^) or continuous (n = 1; Sahle 2020^45^). At protocol stage we misinterpreted the available biostatistical methodology and conversion from continuous to categorical is not possible. Hence, the paper reporting the social health exposure as continuous (Sahle 2020^45^) was excluded from meta‐analysis. Three papers (Strodl 2003,^27^ Strodl 2008,^28^ Simons 2013^40^) remained, with four outcomes, which was sufficient for meta‐analysis.

**FIGURE 2:**
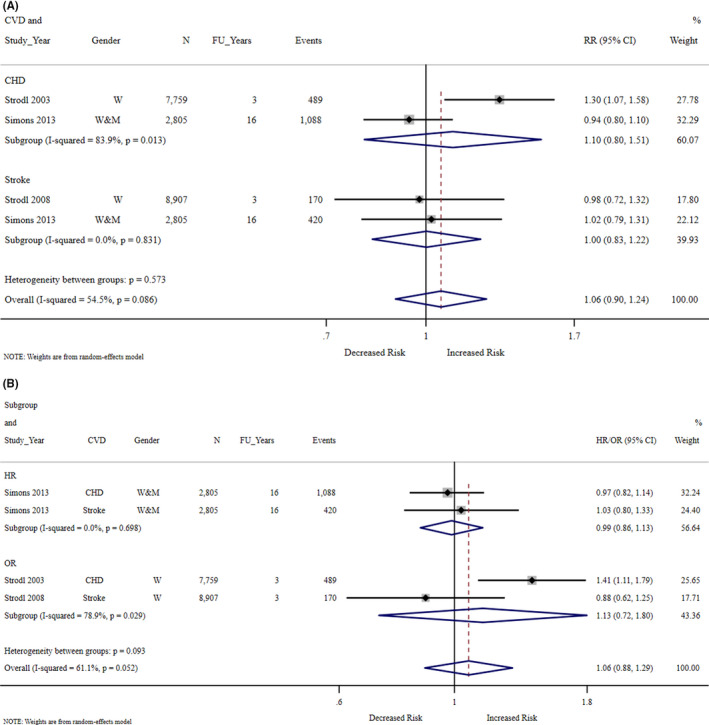
Forest plot of social health as a predictor of cardiovascular disease^a^. ^a^Better social health (defined as ‘good’ or ‘very high & high’) as the reference. FU_Years, follow‐up in years. *I* ^2^ 95% confidence intervals: (A) CHD 0.0%‐96.8%, Stroke 0.0%‐0.0%, Overall 0.0%‐85.6%. (B) HR 0.0%‐0.0%, OR 0.0%‐95.8%, Overall 0.0%‐87.8%. (A) Conversion to relative risk. (B) Estimates as reported in the original papers

For the first meta‐analysis method, we converted odds ratios or hazard ratios into relative risk (Figure 2A), demonstrating no association between social health and CVD. We speculate that including the non‐statistically significant findings from Sahle 2020^45^ (excluded from our meta‐analysis based on exposure assessed as continuous), would not alter our finding. However, caution is required when interpreting Figure 2 due to a number of assumptions that were required to compile these results. Strodl 2003^27^ and Strodl 2008^28^ initially assessed DSSI as three categories, but to make it more comparable to Simons 2013,^40^ we re‐categorised the data into two categories (“very high‐high” vs. “low‐fair”) and therefore unadjusted estimates are presented. Through this process we noticed that in Strodl 2008^28^’s Table 1, the numbers for high DSSI did not align with the odds ratio and speculate that the reference category was incorrectly reverted. As Simons 2013^40^ did not provide the prevalence of those not‐exposed (ie ‘good’) we used the Strodl 2003^27^ and Strodl 2008^28^ estimates for the “very high‐high” category. We chose the longest follow‐up period, which was 16 years for Simons 2013^40^ (who also reported 8 years).

For the second meta‐analysis method, we used the effect estimates reported in the three included studies^27^, ^28^, ^40^ (Figure 2B), which also demonstrated no association between social health and CVD. Again, caution is required regarding interpretation for Strodl 2003^27^ which did not provide the non‐statistically significant adjusted findings, hence the unadjusted statistically significant findings are presented.

Across the four outcomes there was substantial heterogeneity (Figure 2A *I* ^2^ = 54.5%, 95% CI = 0.0%‐85.6%; Figure 2B *I* ^2 ^= 61.1%, 95% CI = 0.0%‐87.8%) but the difference did not reach statistical significance (*P* > .05). There was no strong evidence of publication bias (Appendix D) or small study effects (Egger's test, *P* = .919).

#### 3.4.1 Sub‐analyses

No association was observed regardless of CVD subtype (CHD or stroke) or data source (ALSWH or Dubbo) (Figure 2). Further sub‐analyses were not possible as they would likely reflect data source differences rather than differences by gender, baseline year, or years of follow‐up. No study provided stratification by partner status and all studies recruited people living in Australia.

## 4 DISCUSSION

The five included papers reported six CVD outcomes and represented three Australian longitudinal cohort studies. The included papers reported no association between social health and incidence of CVD in fully adjusted models, and most unadjusted models. Notably, all included papers were assessed as high risk of bias, mainly due to lack of adjustment for important confounders, self‐report of CVD and short follow‐up.

Since our systematic search in June 2020, we have identified only one further publication that would have been included in this review. Freak‐Poli et al^22^ followed 11 486 relatively healthy Australians over the age of 70 years from the ASPirin in Reducing Events in the Elderly (ASPREE) trial for an average of 4.5 years. Overall, older healthy Australians with poor social health (either socially isolated, had low social support or were lonely) were 42% more likely to develop CVD and twice as likely to die from CVD. This was unchanged after accounting for already established CVD risk factors of age, gender, tobacco smoking, systolic blood pressure, high‐density lipoprotein, non‐HDL, diabetes, serum creatinine, and antihypertensive drug use. When looking at the components of social health separately, social isolation (66% greater risk) and low social support (twice the risk), but not loneliness, predicted incidence of CVD. When assessing CVD types separately, all measures of poor social health predicted ischemic stroke, and inconsistent patterns for heart failure hospitalization and myocardial infarction. Freak‐Poli et al^22^ also assessed interaction effects with other CVD risk‐factors and reported that the relationship between poor social health and incidence of CVD was stronger among older Australians who smoked tobacco, lived in a major city, or were aged 70‐75 years (rather than the 75+ years age category). Importantly, CVD was a prespecified secondary endpoint of the main ASPREE trial, and was diagnosed with adjudication by an expert committee. Social isolation and social support were assessed using questions from the Revised Lubben Social Network Scale, while loneliness was assessed through one question from the Center for Epidemiological Studies – Depression (CESD) Scale. While Freak‐Poli et al’s^22^ findings overcome the use of self‐reported CVD, the generalisability is limited to healthy older adults as the participants did not have CVD, dementia, significant physical disability, or any other disease likely to cause death in the next 5 years. Hence further studies assessing the relationship between social health and CVD in Australia and New Zealand are required.

Despite our inconclusive findings, one excluded study provides evidence that the international patterns between poor social health and CVD are relevant to New Zealanders. Caspi et al^46^ and Danese et al^47^ were excluded as their outcome was cardiovascular multifactorial risk status, rather than incidence of CVD. The longitudinal study followed 1037 babies from birth to age 32 years (thus far) and assessed whether social isolation measured at 5, 7, 9, 11 and 26 years was associated with CVD risk.^46^, ^47^ This substantive longitudinal study demonstrated that childhood isolation was associated with adult cardiovascular multifactorial risk status at age 32, independent of other childhood indicators (socioeconomic status, IQ, overweight), health behaviours (physical activity, smoking, alcohol use), and exposure to stressful life events. Social isolation across all ages (childhood, adolescence and adulthood) had a cumulative and dose‐response relationship with adult cardiovascular multifactorial risk at age 26 (RR: 2.58, 95% CI: 1.46‐4.56).^46^ The relationship between social isolation across the life‐course and age‐related disease risk factors in this cohort was also observed at 32 years.^47^

Future research needs to assess the contribution of Australia's and New Zealand's unique geographical spread, which may be masking the relationship between poor social health and incidence of CVD in the included studies. We know that people living in remote areas of Australian are more likely to have prevalent CVD and die from CVD,^48^ however, we are uncertain of how remoteness affects social health in Australia and New Zealand and whether remoteness influences the relationship between poor social health and CVD. International research has identified that several geographic factors are connected to poor social health, including transport disadvantage, reduced access to services and community infrastructure, increased crime, and lower population density.^8^, ^49^, ^50^ International research also suggests that people who live in rural, outer metropolitan fringe or lower socio‐economic locations are at greater risk of social isolation and loneliness.^8^, ^50^ Aligned with international research, the Australian Department of Health and Ageing reported that “*Many people in rural and remote Australia are socially isolated, with less face‐to‐face contact with family, friends and other support networks*”.^51^ However, contradictory to these findings, social isolation among older Australians is highest “*in the largest urban [city] regions and in sparsely populated states and territories*”.^48^, ^52^ Freak‐Poli et al’s^22^ finding support this as the relationship between poor social health and incidence of CVD was stronger among older healthy Australians who lived in a major city, compared to older healthy Australians living in inner regional areas. Other research has suggested that social isolation patterns in Australia seem to go beyond a simple rural/urban divide. “*Across Australia, there was no appreciable difference in the level of social isolation [amongst older Australians] between metropolitan and non‐metropolitan regions …[However], notable variation emerges between regions when the data are mapped*.”^53^ Therefore further research is required to assess social health prevalence based on geographical location, and the implications for CVD risk.

### 4.1 Comparison to international literature

The few papers we included provided findings that contradict the international systematic review reporting that poor social relationships are associated with a 29% and 32% increase in risk of CHD and stroke incidence respectively.^26^ As part of the 23 included papers, Valtorta et al^26^ included two Australian papers (Strodl 2003^27^ and Strodl 2008^28^), which were also included in our review. We are unsure if Simons 2013^40^ was identified by Valtorta et al,^26^ but it was potentially excluded as eligibility criteria was unclear in the short report. From Simons 2013^40^’s referenced paper,^41^ author correspondence^18^, ^25^, ^29^, ^32^ and prior papers,^42^, ^43^, ^44^ we observed that participants with prevalent CHD and stroke at baseline were included in Simons 2013^40^’s sample, and prior events were adjusted for in the main analysis. Hence, inclusion in our review does not differ from Valtorta et al^26^’s inclusion criteria. Byles 2015^39^ and Sahle 2020,^45^ found in our review, were likely published after Valtorta et al^26^’s search date.

There seems to be very little difference between Valtorta et al^26^’s 2016 international systematic review and our current systematic review. Although Valtorta et al^26^ state “*loneliness and social isolation*” in the title, the search terms included “loneliness, social isolation, social relationships, social support, social network”. Valtorta et al^26^ limited the CVD outcomes to CHD and stroke, however, we did not find any additional overall CVD outcome in our broader search. Similarly to our study, Valtorta et al^26^ placed no restriction on the study population, but only longitudinal studies were eligible in order to investigate temporal relationships. Our included papers analysed relatively large samples, initiated across three different decades, which were followed for between 3 and 16 years. Valtorta et al^26^ and our study both included papers which assessed the temporal relationships through logistic regression or cox proportional hazards modelling, and limited the meta‐analyses to papers which assessed the social health exposure as categorical.

We were only able to identify two slight distinctions between our systematic review and Valtorta et al^26^’s international systematic review. First, while both included papers assessing CVD through self‐report (as well as medical diagnosis), the proportion of included papers which used the self‐reported method differed. In our study, all papers relied on baseline self‐report prevalent CVD and only one study (Simons 2013^40^) assessed incidence of CVD through clinical diagnosis. Valtorta et al^26^ reported that four of the 23 included papers relied on self‐reported CVD. Hence, 80% of our included papers relied on self‐reported incidence of CVD data, which is a lot higher than Valtorta et al^26^’s 17%. Second, when meta‐analyses were limited to studies that were in a format suitable for pooling we both removed papers that reported null findings. In our review, we excluded one study (Sahle 2020^45^) from meta‐analysis which accounted for 26% of our CHD events and we speculate that inclusion of this additional null association study would not affect our findings. However, Valtorta et al^26^ excluded four studies from meta‐analyses which were from unique datasets, of which three reported null findings at multivariable adjustment. The null findings were in relatively large samples (n = 2334, n = 4251, and n = 9758) but only accounted for 9% of the CHD events and 3% of the stroke events. The influence of these excluded studies on Valtorta et al^26^’s statistically significant findings is unclear.

### 4.2 Strengths and limitations

Our review mirrored the methodology of an international systematic review,^26^ which allowed direct comparison of findings. Inclusion of longitudinal data provided directional assessment of social health as a risk factor for incidence of CVD, reducing the issue of reverse causation. Conversion of odds ratios and hazard ratios to relative risks allowed direct comparison between studies, however we did misinterpret at protocol stage that biostatistical methodology is not available for conversion from continuous to categorical ratios. We improved upon the protocol by using a specified quality assessment tool for the risk of bias and assessing publication bias. The main limitation was the small number of eligible studies, which came from only three longitudinal data sets, reported three CVD sub‐types (CHD, stroke and heart disease) with no overall CVD outcome, relied heavily on self‐reported CVD data, with only one assessing loneliness, resulting in inconclusive findings and the inability to conduct sub‐group analyses. No studies stratified by gender or partner status, limiting intended reporting. Finally, the data captured in this review was collected prior to the Coronavirus Disease 2019 (COVID‐19) pandemic, which has disrupted people's lifestyle including health behaviours and healthcare delivery. The Cardiac Society of Australia and New Zealand (CSANZ) position statement discusses how the pandemic may have influenced social health in regards to CVD outcomes.^54^

### 4.3 Conclusion

Our systematic review is inconclusive regarding the association between social isolation, lack of social support and loneliness and CVD incidence among people living in Australia and New Zealand. We identified five eligible papers from three longitudinal Australian cohort studies. No eligible studies were identified from New Zealand, highlighting a huge deficit in current research, as even research in Australia may not be generalisable due to different social, political and cultural systems. Included papers reported no association between social health and incidence of CVD in fully adjusted models, and most unadjusted models. The included papers relied heavily on self‐reported CVD prevalence and incidence, hence further studies in Australia using medical diagnosis of CVD (through medical records, death certificates or national registers) are required. Additionally, further research should explore whether Australia's and New Zealand's unique geographical population spread plays a role in the relationship between social health and CVD.

## CONFLICT OF INTEREST

None declared. The data collection, analysis and interpretation of data; the writing of the manuscript; and the decision to submit the manuscript for publication were solely at the discretion of the researchers, independent of funders.

## AUTHOR CONTRIBUTIONS

RF, AZZP and JH contributed to data screening and data extraction. RF and AZZP undertook quality appraisal. RF undertook calculations for meta‐analysis. RF and FB undertook critical interpretation of the data. RF wrote the manuscript. All authors contributed to, as well as, approved the final manuscript.

## APPENDIX A

### Search strategies

(A) Embase from the earliest record to 21st June 2020

(B) Medline from the earliest record to 21st June 2020

(C) Scopus from the earliest record to 21st June 2020

(D) Web of Science from the earliest record to 21st June 2020

## APPENDIX B

### Excluded studies

**Table:**

Study	Title	Authors	DOI	Exclusion reason
Abbott 2010	Barriers and enhancers to dietary behaviour change for Aboriginal people attending a diabetes cooking course	Abbott, P.; Davison, J.; Moore, L.; Rubinstein, R.		03. Not CVD
Abimbola 2019	The NASSS framework for ex post theorisation of technology‐supported change in healthcare: worked example of the TORPEDO programme	Abimbola, S.; Patel, B.; Peiris, D.; Patel, A.; Harris, M.; Usherwood, T.; Greenhalgh, T.	10.1186/s12916‐019‐1463‐x	02. Not social health
Adams 2009	Effects of area deprivation on health risks and outcomes: a multilevel, cross‐sectional, Australian population study	Adams, R. J.; Howard, N.; Tucker, G.; Appleton, S.; Taylor, A. W.; Chittleborough, C.; Gill, T.; Ruffin, R. E.; Wilson, D. H.	10.1007/s00038‐009‐7113‐x	02. Not social health
Albarqouni 2019	External validation and comparison of four cardiovascular risk prediction models with data from the Australian Diabetes, Obesity and Lifestyle study	Albarqouni, L.; Doust, J. A.; Magliano, D.; Barr, E. L. M.; Shaw, J. E.; Glasziou, P. P.	10.5694/mja2.12061	02. Not social health
Alfonso 2012	Perception of worsening health predicts mortality in older men: the Health in Men Study (HIMS)	Alfonso, H.; Beer, C.; Yeap, B. B.; Hankey, G. J.; Flicker, L.; Almeida, O. P.	10.1016/j.archger.2012.04.005	06. Social health not assessed as a predictor of CVD
Al‐Ganmi	Medication adherence and predictive factors in patients with cardiovascular disease: a cross‐sectional study	Al‐Ganmi, A. H. A.; Alotaibi, A.; Gholizadeh, L.; Perry, L.	10.1111/nhs.12681	04. CVD prevalence
Allen 2013	Quality of life impact of cardiovascular and affective conditions among older residents from urban and rural communities	Allen, J.; Inder, K. J.; Harris, M. L.; Lewin, T. J.; Attia, J. R.; Kelly, B. J.	10.1186/1477‐7525‐11‐140	06. Social health not assessed as a predictor of CVD
Allisey 2016	An application of an extended effort‐reward imbalance model to police absenteeism behaviour	Allisey, A.; Rodwell, J.; Noblet, A.	10.1108/pr‐06‐2014‐0125	03. Not CVD
Almeida 2005	Depression and smoking amongst older general practice patients	Almeida, O. P.; Pfaff, J. J.	10.1016/j.jad.2005.02.014	03. Not CVD
Almeida 2011	A practical approach to assess depression risk and to guide risk reduction strategies in later life	Almeida, O. P.; Alfonso, H.; Pirkis, J.; Kerse, N.; Sim, M.; Flicker, L.; Snowdon, J.; Draper, B.; Byrne, G.; Goldney, R.; Lautenschlager, N. T.; Stocks, N.; Scazufca, M.; Huisman, M.; Araya, R.; Pfaff, J.	10.1017/S1041610210001870	06. Social health not assessed as a predictor of CVD
Almeida 2011	Complaints of difficulty to fall asleep increase the risk of depression in later life: the health in men study	Almeida, O. P.; Alfonso, H.; Yeap, B. B.; Hankey, G.; Flicker, L.	10.1016/j.jad.2011.05.045	06. Social health not assessed as a predictor of CVD
Almeida 2012	Cardiovascular disease, depression and mortality: the Health In Men Study	Almeida, O. P.; Alfonso, H.; Flicker, L.; Hankey, G. J.; Norman, P. E.	10.1097/JGP.0b013e318211c1ed	06. Social health not assessed as a predictor of CVD
Almeida 2013	Cardiovascular diseases do not influence the mental health outcome of older men with depression over 6 y	Almeida, O. P.; Alfonso, H.; Yeap, B. B.; Hankey, G. J.; Flicker, L.	10.1016/j.jad.2012.06.043	06. Social health not assessed as a predictor of CVD
Anderson 1995	A population‐based assessment of the impact and burden of caregiving for long‐term stroke survivors	Anderson, C. S.; Linto, J.; Stewart‐Wynne, E. G.		06. Social health not assessed as a predictor of CVD
Anderson 1996	Validation of the Short Form 36 (SF‐36) health survey questionnaire among stroke patients	Anderson, C.; Laubscher, S.; Burns, R.		04. CVD Prevalence
Anderson 2004	The postmodern heart: War veterans' experiences of invasive cardiac technology	Anderson, C. C.	10.1111/j.1365‐2648.2004.02985.x	06. Social health not assessed as a predictor of CVD
Anderson 2006	The effects of a multimodal intervention trial to promote lifestyle factors associated with the prevention of cardiovascular disease in menopausal and postmenopausal Australian women	Anderson, D.; Mizzari, K.; Kain, V.; Webster, J.	10.1080/07399330500506543	06. Social health not assessed as a predictor of CVD
Andrew 2013	Long‐term unmet needs of community dwelling stroke survivors and carers in Australia	Andrew, N. E.; Kilkenny, M.; Naylor, R.; Purvis, T.; Cadilhac, D. A.	10.1159/000353129	03. Not CVD
Andrew 2013	Differences in long‐term unmet needs between younger and older stroke survivors	Andrew, N. E.; Kilkenny, M.; Naylor, R.; Purvis, T.; Cadilhac, D. A.	10.1159/000353129	04. CVD prevalence
Andrew 2013	Understanding the factors associated with unmet needs in Australian stroke survivors	Andrew, N. E.; Kilkenny, M. F.; Naylor, R.; Purvis, T.; Cadilhac, D. A.	10.1111/ijs.12142	04. CVD prevalence
Andrew 2013	Understanding the factors associated with unmet needs in Australian stroke survivors	Andrew, N. E.; Kilkenny, M. F.; Naylor, R.; Purvis, T.; Cadilhac, D. A.	10.1111/ijs.12142	04. CVD prevalence
Andrew 2013	The impact of stroke survivor disability on the long‐term needs of carers	Andrew, N. E.; Kilkenny, M.; Naylor, R.; Purvis, T.; Lalor, E.; Cadilhac, D. A.	10.1111/ijs.12172	04. CVD prevalence
Andrew 2014	Understanding long‐term unmet needs in Australian survivors of stroke	Andrew, N. E.; Kilkenny, M.; Naylor, R.; Purvis, T.; Lalor, E.; Moloczij, N.; Cadilhac, D. A.	10.1111/ijs.12325	06. Social health not assessed as a predictor of CVD
Aoun 2017	Bereavement support for family caregivers: The gap between guidelines and practice in palliative care	Aoun, S. M.; Rumbold, B.; Howting, D.; Bolleter, A.; Breen, L. J.	10.1371/journal.pone.0184750	03. Not CVD
Armstrong 2012	Living with aphasia: three Indigenous Australian stories	Armstrong, E.; Hersh, D.; Hayward, C.; Fraser, J.; Brown, M.	10.3109/17549507.2011.663790	03. Not CVD
Arndt 2009	"Others had similar problems and you were not alone": Evaluation of an open‐group mutual aid model in cardiac rehabilitation	Arndt, M.; Murchie, F.; Schemb, A. M.; Davidson, P. M.	10.1097/JCN.0b013e3181a1c236	04. CVD prevalence
Arroll 2009	Managing cardiovascular risk in the future	Arroll, B.; Elley, C. R.; Fitton, A.; Lebovitz, H.	10.1002/9781444303353.ch12	02. Not social health
Aslani 2011	A community pharmacist delivered adherence support service for dyslipidaemia	Aslani, P.; Rose, G.; Chen, T. F.; Whitehead, P. A.; Krass, I.	10.1093/eurpub/ckq118	03. Not CVD
Aspin 2012	Strategic approaches to enhanced health service delivery for Aboriginal and Torres Strait Islander people with chronic illness: a qualitative study	Aspin, C.; Brown, N.; Jowsey, T.; Yen, L.; Leeder, S.	10.1186/1472‐6963‐12‐143	03. Not CVD
Astley 2011	Health resource variability in the achievement of optimal performance and clinical outcome	Astley, C. M.; MacDougall, C. J.; Davidson, P. M.; Chew, D. P.	10.1161/CIRCOUTCOMES.110.960229	02. Not social health
Attard 2012	The comparative effects of multi‐modality aphasia therapy and constraint‐induced aphasia therapy‐plus treatments for severe chronic Broca's aphasia: a pilot study	Attard, M.; Rose, M.; Lanyon, L.		03. Not CVD
Azzopardi 2009	Health‐related quality of life 2 y after coronary artery bypass graft surgery	Azzopardi, S.; Lee, G.	10.1097/JCN.0b013e31819b2125	06. Social health not assessed as a predictor of CVD
Badcock 2018	Loneliness in psychotic illness and its association with cardiometabolic disorders	Badcock, J. C.; Mackinnon, A.; Waterreus, A.; Watts, G. F.; Castle, D.; McGrath, J. J.; Morgan, V. A.	10.1016/j.schres.2018.09.021	03. Not CVD
Badcock 2019	Loneliness in psychotic illness and its association with cardiometabolic disorders	Badcock, J. C.; Mackinnon, A.; Waterreus, A.; Watts, G. F.; Castle, D.; McGrath, J. J.; Morgan, V. A.	10.1016/j.schres.2018.09.021	03. Not CVD
Bagot 2017	Integrating acute stroke telemedicine consultations into specialists' usual practice: a qualitative analysis comparing the experience of Australia and the United Kingdom	Bagot, K. L.; Cadilhac, D. A.; Bladin, C. F.; Watkins, C. L.; Vu, M.; Donnan, G. A.; Dewey, H. M.; Emsley, H. C. A.; Davies, D. P.; Day, E.; Ford, G. A.; Price, C. I.; May, C. R.; McLoughlin, A. S. R.; Gibson, J. M. E.; Lightbody, C. E.	10.1186/s12913‐017‐2694‐1	06. Social health not assessed as a predictor of CVD
Bailie 2017	Improving preventive health care in Aboriginal and Torres Strait Islander primary care settings	Bailie, J.; Matthews, V.; Laycock, A.; Schultz, R.; Burgess, C. P.; Peiris, D.; Larkins, S.; Bailie, R.	10.1186/s12992‐017‐0267‐z	03. Not CVD
Baker 2019	Risk factors for acute rheumatic fever: literature review and protocol for a case‐control study in New Zealand	Baker, M. G.; Gurney, J.; Oliver, J.; Moreland, N. J.; Williamson, D. A.; Pierse, N.; Wilson, N.; Merriman, T. R.; Percival, T.; Murray, C.; Jackson, C.; Edwards, R.; Page, L. F.; Mow, F. C.; Chong, A.; Gribben, B.; Lennon, D.	10.3390/ijerph16224515	08. Review
Bannink 2006	Web‐based assessment of cardiovascular disease risk in routine primary care practice in New Zealand: the first 18 000 patients (PREDICT CVD‐1)	Bannink, L.; Wells, S.; Broad, J.; Riddell, T.; Jackson, R.		02. Not social health
Barker 2005	Upper limb recovery after stroke: the stroke survivors' perspective	Barker, RN; Brauer, S. G.		04. CVD prevalence
Bean 2007	Ethnic differences in illness perceptions, self‐efficacy and diabetes self‐care	Bean, D.; Cundy, T.; Petrie, K. J.	10.1080/14768320600976240	03. Not CVD
Beard 2009	Influence of socioeconomic and cultural factors on rural health	Beard, J. R.; Tomaska, N.; Earnest, A.; Summerhayes, R.; Morgan, G.	10.1111/j.1440‐1584.2008.01030.x	03. Not CVD
Beckley 2007	The influence of the quality and quantity of social support in the promotion of community participation following stroke	Beckley, MN	10.1111/j.1440‐1630.2007.00643.x	01. Not AUS/NZ
Beesley 2011	Art after stroke: the qualitative experience of community dwelling stroke survivors in a group art programme	Beesley, K.; White, J. H.; Alston, M. K.; Sweetapple, A. L.; Pollack, M.	10.3109/09638288.2011.571333	07. Intervention
Billah 2014	AusSCORE II in predicting 30‐d mortality after isolated coronary artery bypass grafting in Australia and New Zealand	Billah, B.; Huq, M. M.; Smith, J. A.; Sufi, F.; Tran, L.; Shardey, G. C.; Reid, C. M.	10.1016/j.jtcvs.2014.02.027	02. Not social health
Blacker 2010	Evaluation of the effectiveness of an acceptance and commitment therapy (ACT) groupprogram to improve coping and quality of life for individuals post stroke	Blacker, D. J.; Byrnes, M. L.; Beilby, J. M.	10.1111/j.1747‐4949.2010.00458‐4.x	07. Intervention
Blacker 2019	Indigenous stroke care: differences, challenges and a need for change	Blacker, D.; Armstrong, E.	10.1111/imj.14399	04. CVD prevalence
Blomqvist 2018	Enabling healthy living: Experiences of people with severe mental illness in psychiatric outpatient services	Blomqvist, M.; Sandgren, A.; Carlsson, I. M.; Jormfeldt, H.	10.1111/inm.12313	03. Not CVD
Bogomolova 2018	Dietary intervention for people with mental illness in South Australia	Bogomolova, S.; Zarnowiecki, D.; Wilson, A.; Fielder, A.; Procter, N.; Itsiopoulos, C.; O'Dea, K.; Strachan, J.; Ballestrin, M.; Champion, A.; Parletta, N.	10.1093/heapro/daw055	03. Not CVD
Bonner 2019	Implementing cardiovascular disease prevention guidelines to translate evidence‐based medicine and shared decision making into general practice: theory‐based intervention development, qualitative piloting and quantitative feasibility	Bonner, C.; Fajardo, M. A.; Doust, J.; McCaffery, K.; Trevena, L.	10.1186/s13012‐019‐0927‐x	02. Not social health
Booth 1997	Physical activity preferences, preferred sources of assistance, and perceived barriers to increased activity among physically inactive Australians	Booth, M. L.; Bauman, A.; Owen, N.; Gore, C. J.		03. Not CVD
Boult 2011	Developing tools to predict outcomes following cardiovascular surgery	Boult, M.; Fitzpatrick, K.; Barnes, M.; Maddern, G.; Fitridge, R.	10.1111/j.1445‐2197.2010.05644.x	02. Not social health
Bradshaw 2015	Validation study of GRACE risk scores in indigenous and non‐indigenous patients hospitalized with acute coronary syndrome	Bradshaw, P. J.; Katzenellenbogen, J. M.; Sanfilippo, F. M.; Hobbs, M. S. T.; Thompson, P. L.; Thompson, S. C.	10.1186/s12872‐015‐0138‐6	02. Not social health
Bramley 2004	A call to action on Maori cardiovascular health	Bramley, D.; Riddell, T.; Crengle, S.; Curtis, E.; Harwood, M.; Nehua, D.; Reid, P.		02. Not social health
Brauer 2018	Improving physical activity after stroke via treadmill training and self management (IMPACT): a protocol for a randomised controlled trial	Brauer, S. G.; Kuys, S. S.; Paratz, J. D.; Ada, L.	10.1186/s12883‐018‐1015‐6	02. Not social health
Brazionis 2017	An evaluation of the telehealth facilitation of diabetes and cardiovascular care in remote Australian Indigenous communities: protocol for the telehealth eye and associated medical services network [TEAMSnet] project, a pre‐post study design	Brazionis, L.; Jenkins, A.; Keech, A.; Ryan, C.; Bursell, S. E.; T. EAMSnet Study Group	10.1186/s12913‐016‐1967‐4	03. Not CVD
Broad 2007	Zero end‐digit preference in recorded blood pressure and its impact on classification of patients for pharmacologic management in primary care ‐ PREDICT‐CVD‐6	Broad, J.; Wells, S.; Marshall, R.; Jackson, R.	10.3399/096016407782317964	02. Not social health
Brodaty 2007	Rates of depression at 3 and 15 mo poststroke and their relationship with cognitive decline: The Sydney stroke study	Brodaty, H.; Withall, A.; Altendorf, A.; Sachdev, P. S.	10.1097/JGP.0b013e3180590bca	04. CVD prevalence
Brown 2006	Uncovering the determinants of cardiovascular disease among indigenous people	Brown, A. D.; Morrissey, M. J.; Sherwood, J. M.	10.1080/13557850500485485	06. Social health not assessed as a predictor of CVD
Brown 2013	Making a good time": the role of friendship in living successfully with aphasia	Brown, K.; Davidson, B.; Worrall, L. E.; Howe, T.	10.3109/17549507.2012.692814	03. Not CVD
Brundisini 2013	Chronic disease patients' experiences with accessing health care in rural and remote areas: a systematic review and qualitative meta‐synthesis	Brundisini, F.; Giacomini, M.; DeJean, D.; Vanstone, M.; Winsor, S.; Smith, A.		01. Not AUS/NZ
Bulman 2011	Mibbinbah and spirit healing: Fostering safe, friendly spaces for indigenous males in Australia	Bulman, J.; Hayes, R.	10.3149/jmh.1001.6	03. Not CVD
Bunani 2012	The association between social support and psychosocial factors upon mortality and quality of life	Bunani, A. D.; Sedgewick, J. M.; Lehbi, A. M.	10.1111/j.1440‐1797.2012.01633.x	01. Not AUS/NZ
Bunker 2003	"Stress" and coronary heart disease: psychosocial risk factors	Bunker, S. J.; Colquhoun, D. M.; Esler, MD; Hickie, I. B.; Hunt, D.; Jelinek, V. M.; Oldenburg, B. F.; Peach, H. G.; Ruth, D.; Tennant, C. C.; Tonkin, A. M.		08. Review
Burgess 2015	Strengthening cardiovascular disease prevention in remote indigenous communities in Australia's northern territory	Burgess, C. P.; Sinclair, G.; Ramjan, M.; Coffey, P. J.; Connors, C. M.; Katekar, L. V.	10.1016/j.hlc.2014.11.008	07. Intervention
Butland 2019	Health behaviours of rural Australians following percutaneous coronary intervention: a systematic scoping review	Butland, M.; Corones‐Watkins, K.; Evanson, A. D.; Cooke, M.	10.22605/RRH4854	08. Review
Byard 2002	Incapacitation or death of a socially isolated parent or carer could result in the death of dependent children	Byard, R. W.	10.1046/j.1440‐1754.2002.00025.x	03. Not CVD
Byles 2014	Psychological distress and comorbid physical conditions: disease or disability?	Byles, J. E.; Robinson, I.; Banks, E.; Gibson, R.; Leigh, L.; Rodgers, B.; Curryer, C.; Jorm, L.	10.1002/da.22162	06. Social health not assessed as a predictor of CVD
Cadden 2007	Educating nurses about cardiac monitoring in a stroke unit	Cadden, S. M.		02. Not social health
Cadilhac 2017	Improving discharge care: The potential of a new organisational intervention to improve discharge after hospitalisation for acute stroke, a controlled before‐after pilot study	Cadilhac, D. A.; Andrew, N. E.; Stroil Salama, E.; Hill, K.; Middleton, S.; Horton, E.; Meade, I.; Kuhle, S.; Nelson, M. R.; Grimley, R.	10.1136/bmjopen‐2017‐016010	07. Intervention
Cameron 2010	Does cognitive impairment predict poor self‐care in patients with heart failure?	Cameron, J.; Worrall‐Carter, L.; Page, K.; Riegel, B.; Lo, S. K.; Stewart, S.	10.1093/eurjhf/hfq042	04. CVD prevalence
Cameron 2016	Carers' views on patient self‐care in chronic heart failure	Cameron, J.; Rhodes, K. L.; Ski, C. F.; Thompson, D. R.	10.1111/jocn.13124	04. CVD prevalence
Cameron 2016	Psychosocial adjustment of patients living with an internal cardioverter defibrillator	Cameron, J.; Mc Evedy, S.; Lugg, E.; Mariani, J.; Ski, C. F.; Thompson, D. R.; Hammash, M. H.; Moser, D. K.	10.1093/eurheartj/ehw433	04. CVD prevalence
Campbell 1994	Disease, impairment, disability and social handicap: a community based study of people aged 70 y and Over	Campbell, A. J.; Busby, W. J.; Robertson, M. C.; Lum, C. L.; Langlois, J. A.; Morgan, F. C.	10.3109/09638289409166015	06. Social health not assessed as a predictor of CVD
Canuto 2011	Study protocol: a pragmatic randomised controlled trial of a 12‐wk physical activity and nutritional education program for overweight Aboriginal and Torres Strait Islander women	Canuto, K. J.; McDermott, R. A.; Cargo, M.; Esterman, A. J.	10.1186/1471‐2458‐11‐655	03. Not CVD
Cape 1994	The influence of clinical problems, age and social support on outcomes for elderly persons referred to regional aged care assessment teams	Cape, R. D.; Gibson, S. J.		03. Not CVD
Caperchione 2011	Physical activity behaviours of Culturally and Linguistically Diverse (CALD) women living in Australia: a qualitative study of socio‐cultural influences	Caperchione, C. M.; Kolt, G. S.; Tennent, R.; Mummery, W. K.	10.1186/1471‐2458‐11‐26	03. Not CVD
Caspi 2006	Socially isolated children 20 y later ‐ risk of cardiovascular disease	Caspi, A.; Harrington, H.; Moffitt, T. E.; Milne, B. J.; Poulton, R.	10.1001/archpedi.160.8.805	03. Not CVD
Cassel 1974	Hypertension and cardiovascular disease in migrants: a potential source of clues?	Cassel, J.		06. Social health not assessed as a predictor of CVD
Chan 1285	Ethnic and socioeconomic disparities in the prevalence of cardiovascular disease in New Zealand	Chan, W. C.; Wright, C.; Riddell, T.; Wells, S.; Kerr, A. J.; Gala, G.; Jackson, R.		02. Not social health
Chan 2008	Ethnic and socioeconomic disparities in the prevalence of cardiovascular disease in New Zealand	Chan, W. C.; Wright, C.; Riddell, T.; Wells, S.; Kerr, A. J.; Gala, G.; Jackson, R.	10.1136/bmj.39455.596181.25	04. CVD prevalence
Cheok 2003	Identification, course, and treatment of depression after admission for a cardiac condition: Rationale and patient characteristics for the Identifying Depression As a Comorbid Condition (IDACC) project	Cheok, F.; Schrader, G.; Banham, D.; Marker, J.; Hordacre, A. L.	10.1016/s0002‐8703(03)00481‐2	04. CVD prevalence
Clark 1998	The effects of depression and abnormal illness behaviour on outcome following rehabilitation from stroke	Clark, M. S.; Smith, D. S.	10.1191/026921598669567216	02. Not social health
Clark 1999	Psychological correlates of outcome following rehabilitation from stroke	Clark, M. S.; Smith, D. S.	10.1191/026921599673399613	02. Not social health
Clark 1999	Changes in family functioning for stroke rehabilitation patients and their families	Clark, M. S.; Smith, D. S.	10.1097/00004356‐199909000‐00003	06. Social health not assessed as a predictor of CVD
Clark 2003	A randomized controlled trial of an education and counselling intervention for families after stroke	Clark, M. S.; Rubenach, S.; Winsor, A.		07. Intervention
Clark 2010	Home based cardiac rehabilitation	Clark, A. M.	10.1136/bmj.b5510	08. Review
Clark 2015	Development and feasibility testing of an education program to improve knowledge and self‐care among Aboriginal and Torres Strait Islander patients with heart failure	Clark, R. A.; Fredericks, B.; Buitendyk, N. J.; Adams, M. J.; Howie‐Esquivel, J.; Dracup, K. A.; Berry, N. M.; Atherton, J.; Johnson, S.		02. Not social health
Cleland 2010	Individual, social and environmental correlates of physical activity among women living in socioeconomically disadvantaged neighbourhoods	Cleland, V.; Ball, K.; Hume, C.; Timperio, A.; King, A. C.; Crawford, D.	10.1016/j.socscimed.2010.02.028	03. Not CVD
Courtney 2009	Fewer emergency readmissions and better quality of life for older adults at risk of hospital readmission: a randomized controlled trial to determine the effectiveness of a 24‐wk exercise and telephone follow‐up program	Courtney, M.; Edwards, H.; Chang, A.; Parker, A.; Finlayson, K.; Hamilton, K.	10.1111/j.1532‐5415.2009.02138.x	07. Intervention
Cruice 2010	Health‐related quality of life in people with aphasia: Implications for fluency disorders quality of life research	Cruice, M.; Worrall, L.; Hickson, L.	10.1016/j.jfludis.2010.05.008	03. Not CVD
Daly 2000	Health status, perceptions of coping, and social support immediately after discharge of survivors of acute myocardial infarction	Daly, J.; Elliott, D.; Cameron‐Traub, E.; Salamonson, Y.; Davidson, P.; Jackson, D.; Chin, C.; Wade, V.		06. Social health not assessed as a predictor of CVD
Danese 2009	Adverse childhood experiences and adult risk factors for age‐related disease: depression, inflammation, and clustering of metabolic risk markers	Danese, A.; Moffitt, T. E.; Harrington, H.; Milne, B. J.; Polanczyk, G.; Pariante, C. M.; Poulton, R.; Caspi, A.	10.1001/archpediatrics.2009.214	03. Not CVD
Daniel 2011	Environmental risk conditions and pathways to cardiometabolic diseases in indigenous populations	Daniel, M.; Lekkas, P.; Cargo, M.; Stankov, I.; Brown, A.	10.1146/annurev.publhealth.012809.103557	08. Review
Davidson 2003	Identifying the communication activities of older people with aphasia: evidence from naturalistic observation	Davidson, B.; Worrall, L.; Hickson, L.		03. Not CVD
Davidson 2004	Integrated, collaborative palliative care in heart failure: the St. George Heart Failure Service experience 1999‐2002	Davidson, P. M.; Paull, G.; Introna, K.; Cockburn, J.; Davis, J. M.; Rees, D.; Gorman, D.; Magann, L.; Lafferty, M.; Dracup, K.		06. Social health not assessed as a predictor of CVD
Davidson 2005	Activities of home‐based heart failure nurse specialists: a modified narrative analysis	Davidson, P.; Paull, G.; Rees, D.; Daly, J.; Cockburn, J.		04. CVD Prevalence
Davidson 2006	Social communication in older age: lessons from people with aphasia	Davidson, B.; Worrall, L.; Hickson, L.	10.1310/0GGQ‐CJDX‐N2BR‐W7W4	03. Not CVD
Davidson 2008	Social participation for older people with aphasia: the impact of communication disability on friendships	Davidson, B.; Howe, T.; Worrall, L.; Hickson, L.; Togher, L.	10.1310/tsr1504‐325	03. Not CVD
Dean 2009	Exercise intervention to prevent falls and enhance mobility in community dwellers after stroke: a protocol for a randomised controlled trial	Dean, C. M.; Rissel, C.; Sharkey, M.; Sherrington, C.; Cumming, R. G.; Barker, RN; Lord, S. R.; O'Rourke, S. D.; Kirkham, C.	10.1186/1471‐2377‐9–38	07. Intervention
Dean 2014	Treadmill training provides greater benefit to the subgroup of community‐dwelling people after stroke who walk faster than 0.4 m/s: a randomised trial	Dean, C. M.; Ada, L.; Lindley, R. I.	10.1016/j.jphys.2014.03.004	07. Intervention
Dengler 2011	The heart beads program	Dengler, K. A.; Scarfe, G.; Redshaw, S.; Wilson, V.	10.1111/j.1744‐6155.2010.00273.x	04. CVD prevalence
Denham 2019	"This is our life now. our new normal": a qualitative study of the unmet needs of carers of stroke survivors	Denham, A.; Wynne, O.; Baker, A. L.; Spratt, N. J.; Turner, A.; Magin, P.; Janssen, H.; English, C.; Loh, M.; Bonevski, B.	10.1177/1747493019858233	04. CVD prevalence
DiBenedetto 2010	A biopsychosocial model for depressive symptoms following acute coronary syndromes	Di Benedetto, M.; Burns, G. L.; Lindner, H.; Kent, S.	10.1080/08870440903019535	04. CVD prevalence
Dollard 2004	Broadening the reach of cardiac rehabilitation to rural and remote Australia	Dollard, J.; Smith, J.; R. Thompson D; Stewart, S.		08. Review
D'Onise 2012	Does an early childhood intervention affect cardiometabolic risk in adulthood? Evidence from a longitudinal study of preschool attendance in South Australia	D'Onise, K.; Lynch, J. W.; McDermott, R. A.	10.1016/j.puhe.2012.04.012	03. Not CVD
Dracup 2006	A nursing intervention to reduce prehospital delay in acute coronary syndrome: a randomized clinical trial	Dracup, K.; McKinley, S.; Riegel, B.; Mieschke, H.; Doering, L. V.; Moser, D. K.		07. Intervention
Draper 2007	Stress in caregivers of aphasic stroke patients: a randomized controlled trial	Draper, B.; Bowring, G.; Thompson, C.; Thompson, C.; Van Heyst, J.; Conroy, P.; Thompson, J.	10.1177/0269215506071251	07. Intervention
DuPlessis 2018	Traversing the liminal: what can Fontan adults' transition experiences and perspectives teach us about optimizing healthcare?	Du Plessis, K.; Peters, R.; Culnane, E.; D'Udekem, Y.	10.1515/ijamh‐2018‐0020	04. CVD prevalence
Dyall 2008	Stroke: A picture of health disparities in New Zealand	Dyall, L.; Feigin, V.; Brown, P.; Roberts, M.		04. CVD prevalence
Essue 2010	Informal care and the self‐management partnership: implications for Australian health policy and practice	Essue, B. M.; Jowsey, T.; Jeon, Y. H.; Mirzaei, M.; Pearce‐Brown, C. L.; Aspin, C.; Usherwood, T. P.	10.1071/AH09795 10.1207/S15324796ABM2601‐01	04. CVD prevalence
Fernandez 2008	Cardiac rehabilitation coordinators' perceptions of patient‐related barriers to implementing cardiac evidence‐based guidelines	Fernandez, R. S.; Davidson, P.; Griffiths, R.	10.1097/01.JCN.0000317450.64778.a0	04. CVD prevalence
Fernandez 2008	Sociodemographic predictors and reasons for participation in an outpatient cardiac rehabilitation programme following percutaneous coronary intervention	Fernandez, R. S.; Salamonson, Y.; Griffiths, R.; Juergens, C.; Davidson, P.	10.1111/j.1440‐172X.2008.00685.x	04. CVD prevalence
Ferry 2004	Towards a safer culture: clinical pathways in acute coronary syndromes and stroke	Ferry, C. T.; Fitzpatrick, M. A.; Long, P. W.; Levi, C. R.; Bishop, R. O.		02. Not social health
Field 2002	A case study in strategic change: developing a strategic research program to address cardiovascular disease and related disorders in aboriginal and Torres Strait Islander peoples and rural and remote settings	Field, P.; Wakerman, J.		02. Not social health
Finch 2017	Undetected and underserved: the untold story of patients who had a minor stroke: equity of access is particularly concerning for minor stroke	Finch, E. C.; Foster, M. M.; Fleming, J.; Aitken, P. D.; Williams, I.; Cruwys, T.; Worrall, L.	10.5694/mja16.01009	08. Review
Fini 2014	How physically active are people following stroke?	Fini, N. A.; Holland, A. E.; Keating, J.; Simek, J.; Bernhardt, J.	10.1111/ijs.12297	08. Review
Fullagar 2003	Governing women's active leisure: the gendered effects of calculative rationalities within Australian health policy	Fullagar, S. P.	10.1080/0958159031000100206	03. Not CVD
Gallagher 2003	Effects of a telephone counseling intervention on psychosocial adjustment in women following a cardiac event	Gallagher, R.; McKinley, S.; Dracup, K.		07. Intervention
Gallagher 2012	Weight management issues and strategies for people with high cardiovascular risk undertaking an Australian weight loss program: a focus group study	Gallagher, R.; Kirkness, A.; Armari, E.; Davidson, P. M.	10.1111/j.1442‐2018.2011.00651.x	03. Not CVD
Gallagher 2016	Quality of life, social support and cognitive impairment in heart failure patients without diagnosed dementia	Gallagher, R.; Sullivan, A.; Burke, R.; Hales, S.; Sharpe, P.; Tofler, G.	10.1111/ijn.12402	04. CVD prevalence
Garcia 2019	The roles of dispositional coping style and social support in helping people with respiratory disease cope with a breathlessness crisis	Garcia, M. V.; Luckett, T.; Johnson, M.; Hutchinson, A.; Lal, S.; Phillips, J. L.	10.1111/jan.14039	03. Not CVD
Garofalo 2012	Pre‐hospital delay in acute coronary syndromes: PREDICT CVD‐18	Garofalo, D.; Grey, C.; Lee, M.; Exeter, D.; Kerr, A. J.		02. Not social health
Gaskin 2015	Parents experiences of going home with their infant following first stage cardiac surgery for single ventricle heart condition	Gaskin, K. L.; Hutchinson, S.	10.1136/archdischild‐2015‐308599.20	01. Not AUS/NZ
Gaskin 2015	Transition from hospital to home: psychosocial adaptation and adjustment in parents of infants with single ventricle heart conditions	Gaskin, K. L.; Hutchinson, S.	10.1136/archdischild‐2015‐308599.11	01. Not AUS/NZ
Gassner 2003	Aerobic exercise and the post myocardial infarction patient: a review of the literature	Gassner, L. A.; Dunn, S.; Piller, N.	10.1016/S0147‐9563%2803%2900039‐6	02. Not social health
Gill 2016	Feeling angry about current health status: using a population survey to determine the association with demographic, health and social factors	Gill, T. K.; Price, K.; Dal Grande, E.; Daly, A.; Taylor, A. W.	10.1186/s12889‐016‐3232‐5	03. Not CVD
Gliksman 1995	Social support, marital status and living arrangement correlates of cardiovascular disease risk factors in the elderly	Gliksman, MD; Lazarus, R.; Wilson, A.; Leeder, S. R.	10.1016/0277‐9536%2894%2900149‐N	03. Not CVD
Glozier 2013	Psychosocial risk factors for coronary heart disease	Glozier, N.; Tofler, G. H.; Colquhoun, D. M.; Bunker, S. J.; Clarke, D. M.; Hare, D. L.; Hickie, I. B.; Tatoulis, J.; Thompson, D. R.; Wilson, A.; Branagan, M. G.		06. Social health not assessed as a predictor of CVD
Glozier 2014	The national heart foundation of Australia consensus statement on psychosocial risk factors for coronary heart disease	Glozier, N.; Tofler, G. H.; Colquhoun, D. M.; Bunker, S. J.; Clarke, D. M.; Hare, D. L.; Hickie, I. B.; Tatoulis, J.; Thompson, D. R.; Branagan, M.	10.1016/j.gheart.2014.03.1930	06. Social health not assessed as a predictor of CVD
Graven 2011	From rehabilitation to recovery: protocol for a randomised controlled trial evaluating a goal‐based intervention to reduce depression and facilitate participation post‐stroke	Graven, C.; Brock, K.; Hill, K.; Ames, D.; Cotton, S.; Joubert, L.	10.1186/1471‐2377‐11‐73	07. Intervention
Graves 2009	Cost‐effectiveness of an intervention to reduce emergency re‐admissions to hospital among older patients	Graves, N.; Courtney, M.; Edwards, H.; Chang, A.; Parker, A.; Finlayson, K.	10.1371/journal.pone.0007455	07. Intervention
Grey 2010	A comparative analysis of cardiovascular disease risk profiles of five Pacific ethnic groups assessed in New Zealand primary care practice: PREDICT CVD‐13	Grey, C.; Wells, S.; Riddell, T.; Pylypchuk, R.; Marshall, R.; Drury, P.; Elley, R.; Ameratunga, S.; Gentles, D.; Erick‐Peleti, S.; Bell, F.; Kerr, A.; Jackson, R.		02. Not social health
Grey 2010	A comparative analysis of the cardiovascular disease risk factor profiles of Pacific peoples and Europeans living in New Zealand assessed in routine primary care: PREDICT CVD‐11	Grey, C.; Wells, S.; Riddell, T.; Kerr, A.; Gentles, D.; Pylypchuk, R.; Marshall, R.; Ameratunga, S.; Drury, P.; Elley, R.; Kyle, C.; Exeter, D.; Jackson, R.		02. Not Social Health
Grohn 2012	The first 3‐mo post‐stroke: what facilitates successfully living with aphasia?	Grohn, B.; Worrall, L. E.; Simmons‐Mackie, N.; Brown, K.	10.3109/17549507.2012.692813	03. Not CVD
Gu 2016	Identifying ehealth opportunities to support medication adherence ‐ findings of a focus group study	Gu, Y.; Kennely, J.; Warren, J.; Ahn, A. B.; Harwood, M.; Neuwelt, P.; Ammenwerth, E.; Schreier, G.; Horbst, A.; Hayn, D.	10.3233/978‐1‐61499‐645‐3–150	04. CVD prevalence
Hagan 2007	Financial, family, and social factors impacting on cardiac rehabilitation attendance	Hagan, N. A.; Botti, M. A.; Watts, R. J.		04. CVD Prevalence
Hakkennes 2012	Selection for inpatient rehabilitation following severe stroke: an observational study	Hakkennes, S.; Brock, K.; Hill, K.; Churilov, L.	10.1177/1545968312449454	04. CVD prevalence
Hall 2015	Improving longer term outcomes post stroke: exploring the barriers and facilitators that influence unmet need, life quality and participation after stroke	Hall, J.; Hawkins, R.; Dickerson, J.; Crocker, T.; McEachan, R.; Forster, A.	10.1111/ijs.12585	01. Not AUS/NZ
Hammash 2019	Perceived control and quality of life among recipients of implantable cardioverter defibrillator	Hammash, M.; McEvedy, S. M.; Wright, J.; Cameron, J.; Miller, J.; Ski, C. F.; Thompson, D. R.; Biddle, M. J.; Wimsatt, A.; Schrader, M.; Smith, R. V.; Chung, M. L.; Moser, D. K.	10.1016/j.aucc.2018.08.005	04. CVD prevalence
Hammond 2008	Factors associated with persistent risk of depression in older people following discharge from an acute cardiac unit	Hammond, A. J.; Yu, S.; Esa, K.; Jabbour, J.; Wakefield, L.; Ryan, P.; Visvanathan, R.	10.1017/S1041610208007138	04. CVD Prevalence
Hancock 2017	Rational clinical evaluation of suspected acute coronary syndromes: the value of more information	Hancock, D. G.; Chuang, M. Y. A.; Bystrom, R.; Halabi, A.; Jones, R.; Horsfall, M.; Cullen, L.; Parsonage, W. A.; Chew, D. P.	10.1111/1742‐6723.12819	02. Not social health
Hand 1996	Older adults with lifelong intellectual handicap in New Zealand: prevalence, disabilities and implications for regional health authorities	Hand, J. E.; Reid, P. M.		03. Not CVD
Harris 2010	How do we manage patients who become unemployed?	Harris, M. F.; Harris, E.; Shortus, T. D.		03. Not CVD
Hawkes 2009	Randomised controlled trial of a secondary prevention program for myocardial infarction patients ('ProActive Heart'): study protocol. Secondary prevention program for myocardial infarction patients	Hawkes, A. L.; Atherton, J.; Barr, C. B.; Scuffham, P.; Eadie, K.; Miller, N. H.; Oldenburg, B.	10.1186/1471‐2261‐9‐16	07. Intervention
Hawkes 2013	Predictors of physical and mental health‐related quality of life outcomes among myocardial infarction patients	Hawkes, A. L.; Patrao, T. A.; Ware, R.; Atherton, J. J.; Taylor, C. B.; Oldenburg, B. F.	10.1186/1471‐2261‐13‐69	04. CVD prevalence
Hawthorne 2008	Perceived social isolation in a community sample: its prevalence and correlates with aspects of peoples' lives	Hawthorne, G.	10.1007/s00127‐007‐0279‐8	03. Not CVD
Haynes 2019	Community‐based participatory action research on rheumatic heart disease in an Australian Aboriginal homeland: evaluation of the 'On track watch' project	Haynes, E.; Marawili, M.; Marika, B. M.; Mitchell, A. G.; Phillips, J.; Bessarab, D.; Walker, R.; Cook, J.; Ralph, A. P.	10.1016/j.evalprogplan.2019.02.010	02. Not social health
Hayward 2013	Factors influencing the consultants' decision to admit a stroke survivor to and then continue or cease inpatient stroke rehabilitation: a statewide survey	Hayward, K. S.; Aitkin, P. D.; Barker, R. N.; Brauer, S. G.	10.1111/ijs.12143	04. CVD prevalence
Hayward 2014	Admission to and continuation of inpatient stroke rehabilitation in Queensland, Australia: a survey of factors that contribute to the consultant's decision	Hayward, K. S.; Aitken, P. D.; Barker, RN; Brauer, S. G.	10.1017/BrImp.2014.12	04. CVD prevalence
Hein 2013	Myocardial infarction in singapore: ethnic variation in evidence‐based therapy and its association with socioeconomic status, social network size and perceived stress level	Hein, T.; Loo, G.; Tai, B. C.; Phua, Q. H.; Chan, M. Y.; Poh, K. K.; Chia, B. L.; Richards, M.; Lee, C. H.	10.1016/j.hlc.2013.04.119	01. Not AUS/NZ
Hepburn‐Brown 2019	Early decision‐making in acute pulmonary embolism: a retrospective clinical audit	Hepburn‐Brown, M.; Irving, L.; Hammerschlag, G.	10.1111/imj.14042	02. Not social health
Hodge 2013	Social connectedness and predictors of successful ageing	Hodge, A. M.; English, D. R.; Giles, G. G.; Flicker, L.	10.1016/j.maturitas.2013.05.002	03. Not CVD
Horwood 2015	Examining motivations and barriers for attending maintenance community‐based cardiac rehabilitation using the health‐belief model	Horwood, H.; Williams, M. J. A.; Mandic, S.	10.1016/j.hlc.2015.03.023	04. CVD prevalence
Howe 2012	'You needed to rehab… families as well': family members' own goals for aphasia rehabilitation	Howe, T.; Davidson, B.; Worrall, L.; Hersh, D.; Ferguson, A.; Sherratt, S.; Gilbert, J.		03. Not CVD
Hsu‐Hage 2001	A qualitative investigation into the use of health services among Melbourne Chinese	Hsu‐Hage, B. H. H.; Tang, K. C.; Li, R. J.; Lin, V.; Chow, T.; Thien, F.		03. Not CVD
Hua 2017	Validation and recalibration of the Framingham cardiovascular disease risk models in an Australian Indigenous cohort	Hua, X.; McDermott, R.; Lung, T.; Wenitong, M.; Tran‐Duy, A.; Li, M.; Clarke, P.	10.1177/2047487317722913	02. Not social health
Huffman 2010	Cardiovascular health in indigenous communities: successful programs	Huffman, MD; Galloway, J. M.	10.1016/j.hlc.2010.02.013	04. CVD prevalence
Hutchinson 2015	Relationship between health‐related quality of life, comorbidities and acute health care utilisation, in adults with chronic conditions	Hutchinson, A. F.; Graco, M.; Rasekaba, T. M.; Parikh, S.; Berlowitz, D. J.; Lim, W. K.	10.1186/s12955‐015‐0260‐2	06. Social health not assessed as a predictor of CVD
Hyun 2017	Gender inequalities in cardiovascular risk factor assessment and management in primary healthcare	Hyun, K. K.; Redfern, J.; Patel, A.; Peiris, D.; Brieger, D.; Sullivan, D.; Harris, M.; Usherwood, T.; MacMahon, S.; Lyford, M.; Woodward, M.	10.1136/heartjnl‐2016‐310216	02. Not social health
Ilett 2010	Selecting patients for rehabilitation after acute stroke: are there variations in practice?	Ilett, P. A.; Brock, K. A.; Graven, C. J.; Cotton, S. M.	10.1016/j.apmr.2009.11.028	04. CVD prevalence
Ingles 2007	Sudden cardiac death in the young: a clinical genetic approach	Ingles, J.; Semsarian, C.		02. Not social health
Ingles 2014	Medication non‐compliance in patients with hypertrophic cardiomyopathy	Ingles, J.; Johnson, R.; Driscoll, E.; Sarina, T.; Semsarian, C.	10.1177/1474515114521363	04. CVD prevalence
Iyngkaran 2016	Self managing heart failure in remote australia ‐ translating concepts into clinical practice	Iyngkaran, P.; Toukhsati, S. R.; Harris, M.; Connors, C.; Kangaharan, N.; Ilton, M.; Nagel, T.; Moser, D. K.; Battersby, M.	10.2174/1573403x12666160703183001	08. Review
Jackson 2017	Psychosocial screening and assessment practice within cardiac rehabilitation: a survey of cardiac rehabilitation coordinators in Australia	Jackson, A. C.; Le Grande, M. R.; Higgins, R. O.; Rogerson, M.; Murphy, B. M.	10.1016/j.hlc.2016.04.018	04. CVD prevalence
Jacobs 2011	Does being elderly and living alone impact on outcomes following participation in a cardiac rehabilitation program?	Jacobs, D.; Young‐Whitford, A.; Campbell, N.; Chong, S.; Jalaludin, B.; Davidson, P.; Quinn, W.	10.1016/j.hlc.2011.05.606	04. CVD prevalence
Jeacocke 2002	Adopting guideline review criteria as part of a regional project to improve heart failure management in general practice	Jeacocke, D.; Sprogis, A.; Lowe, J.; Heller, R.	10.1108/14664100210427615	02. Not social health
Jeon 2010	Achieving a balanced life in the face of chronic illness	Jeon, Y. H.; Jowsey, T.; Yen, L.; Glasgow, N. J.; Essue, B.; Kljakovic, M.; Pearce‐Brown, C.; Mirzaei, M.; Usherwood, T.; Jan, S.; Kraus, S. G.; Aspin, C.		03. Not CVD
Jeremy 2010	Improving cardiovascular care for indigenous populations	Jeremy, R.; Tonkin, A.; White, H.; Riddell, T.; Brieger, D.; Walsh, W.; Zeitz, C.; Brown, A.; Kritharides, L.	10.1016/j.hlc.2010.02.015	08. Review
Kahl 2016	Quality of life in adults with congenital heart disease: What matters?	Kahl, K. G.; Westhoff‐Bleck, M.	10.21037/jtd.2016.10.66	02. Not social health
Karageorge 2020	Previous experience and walking capacity predict community outings after stroke: an observational study	Karageorge, A.; Vargas, J.; Ada, L.; Kelly, P. J.; McCluskey, A.	10.1080/09593985.2018.1484829	04. CVD prevalence
KarataÅŸ 2017	Perceived social support and psychosocial adjustment in patients with coronary heart disease	KarataÅŸ, T.; BostanoÄŸlu, H.	10.1111/ijn.12558	01. Not AUS/NZ
Kendall 2007	Recovery following stroke: the role of self‐management education	Kendall, E.; Catalano, T.; Kuipers, P.; Posner, N.; Buys, N.; Charker, J.	10.1016/j.socscimed.2006.09.012	07. Intervention
Kenealy 2012	A 'whole of system' approach to compare options for CVD interventions in Counties Manukau	Kenealy, T.; Rees, D.; Sheridan, N.; Moffitt, A.; Tibby, S.; Homer, J.	10.1111/j.1753‐6405.2011.00812.x	02. Not social health
Kennedy 2012	Factors influencing selection for rehabilitation after stroke: a questionnaire using case scenarios to investigate physician perspectives and level of agreement	Kennedy, G. M.; Brock, K. A.; Lunt, A. W.; Black, S. F.	10.1016/j.apmr.2011.11.036	04. CVD prevalence
Kerr 2019	A unified national cardiovascular disease (CVD) risk generator is required to address equity in the management of CVD risk in clinical practice in New Zealand	Kerr, A.; Wells, S.; Moffitt, A.; Lund, M.; Kreichbaum, J.; Harwood, M.; Jackson, R.		02. Not social health
Killey 2014	Paths to work after stroke in Australia	Killey, J.; Gustafsson, L.; Hoyle, M.	10.1017/BrImp.2014.18	02. Not social health
Kiropoulos 2012	Increased psychosocial stress in Greek‐born immigrants compared to Anglo‐Australians with coronary heart disease: the healthy heart, healthy mind study	Kiropoulos, L. A.; Meredith, I.; Tonkin, A.; Clarke, D.; Antonis, P.; Plunkett, J.	10.1016/j.hlc.2012.07.018	06. Social health not assessed as a predictor of CVD
Knight 2017	Developing a synthetic national population to investigate the impact of different cardiovascular disease risk management strategies: a derivation and validation study	Knight, J.; Wells, S.; Marshall, R.; Exeter, D.; Jackson, R.	10.1371/journal.pone.0173170	02. Not social health
Korda 2017	Variation in readmission and mortality following hospitalisation with a diagnosis of heart failure: prospective cohort study using linked data	Korda, R. J.; Du, W.; Day, C.; Page, K.; Macdonald, P. S.; Banks, E.	10.1186/s12913‐017‐2152‐0	04. CVD prevalence
Kowal 2010	Enduring dilemmas of Indigenous health. "You're always hearing about the stats… death happens so often": new perspectives on barriers to Aboriginal participation in cardiac rehabilitation. Comment	Kowal, E. E.; Paradies, Y. C.		04. CVD prevalence
Kritharides 2010	Overview and determinants of cardiovascular disease in indigenous populations	Kritharides, L.; Brown, A.; Brieger, D.; Ridell, T.; Zeitz, C.; Jeremy, R.; Tonkin, A.; Walsh, W.; White, H.	10.1016/j.hlc.2010.02.017	06. Social health not assessed as a predictor of CVD
Lanyon 2018	Exploring participant perspectives of community aphasia group participation: from "I know where I belong now" to "Some people didn't really fit in"	Lanyon, L.; Worrall, L.; Rose, M.	10.1080/02687038.2017.1396574	03. Not CVD
Lauckner 2016	Peer support for people with chronic conditions in rural areas: a scoping review	Lauckner, H. M.; Hutchinson, S. L.		03. Not CVD
Lauder 2006	A comparison of health behaviours in lonely and non‐lonely populations	Lauder, W.; Mummery, K.; Jones, M.; Caperchione, C.	10.1080/13548500500266607	03. Not CVD
Lauder 2006	Social capital, age and religiosity in people who are lonely	Lauder, W.; Mummery, K.; Sharkey, S.		03. Not CVD
Lawrence 2017	Yoga for stroke rehabilitation	Lawrence, M.; Celestino, F. T.; Matozinho, H. H. S.; Govan, L.; Booth, J.; Beecher, J.	10.1002/14651858.CD011483.pub2	07. Intervention
Leigh 2004	The clinical support systems program	Leigh, J. A.; Long, P. W.; Phillips, P. A.; Mortimer, R. H.		03. Not CVD
Leung 2010	Geographic issues in cardiac rehabilitation utilization: a narrative review	Leung, Y. W.; Brual, J.; Macpherson, A.; Grace, S. L.	10.1016/j.healthplace.2010.08.004	01. Not AUS/NZ
Li 2016	Impact of socioeconomic and risk factors on cardiovascular disease and type II diabetes in Australia: comparison of results from longitudinal and cross‐sectional designs	Li, J. J.; Kinfu, Y.	10.1136/bmjopen‐2015‐010 215	02. Not social health
Liu 2016	Risk factors for obstructive sleep apnea are prevalent in people with psychosis and correlate with impaired social functioning and poor physical health	Liu, D.; Myles, H.; Foley, D. L.; Watts, G. F.; Morgan, V. A.; Castle, D.; Waterreus, A.; Mackinnon, A.; Galletly, C. A.	10.3389/fpsyt.2016.00139	06. Social health not assessed as a predictor of CVD
Lo 2012	Pediatric stroke outcome measure predicts cognitive and functional deficits after childhood ischemic stroke	Lo, W.; Gordon, A.; Greenham, M.; Gomes, A.; Hajek, C.; Mackay, M.; Yeates, K. O.; Anderson, V.		04. CVD prevalence
Lobstein 2004	Obesity in children and young people: a crisis in public health	Lobstein, T.; Baur, L.; Uauy, R.		03. Not CVD
Longman 2012	Frequent hospital admission of older people with chronic disease: a cross‐sectional survey with telephone follow‐up and data linkage	Longman, J. M.; I Rolfe, M.; Passey, MD; Heathcote, K. E.; Ewald, D. P.; Dunn, T.; Barclay, L. M.; Morgan, G. G.	10.1186/1472‐6963‐12‐373	06. Social health not assessed as a predictor of CVD
Lord 2008	How feasible is the attainment of community ambulation after stroke? A pilot randomized controlled trial to evaluate community‐based physiotherapy in subacute stroke	Lord, S.; McPherson, K. M.; McNaughton, H. K.; Rochester, L.; Weatherall, M.	10.1177/0269215507081922	07. Intervention
Lovell 2010	Telehealth technologies for managing chronic disease ‐ experiences from Australia and the UK	Lovell, N. H.; Redmond, S. J.; Basilakis, J.; Shany, T.; Celler, B. G.	10.1109/IEMBS.2010.5626312	02. Not social health
Lynch 2016	Education‐only versus a multifaceted intervention for improving assessment of rehabilitation needs after stroke; a cluster randomised trial	Lynch, E. A.; Cadilhac, D. A.; Luker, J. A.; Hillier, S. L.	10.1186/s13012‐016‐0487‐2	02. Not social health
Lynch 2018	Activity monitors for increasing physical activity in adult stroke survivors	Lynch, E. A.; Jones, T. M.; Simpson, D. B.; Fini, N. A.; Kuys, S. S.; Borschmann, K.; Kramer, S.; Johnson, L.; Callisaya, M. L.; Mahendran, N.; Janssen, H.; English, C.	10.1002/14651858.CD012543.pub2	07. Intervention
Macniven 2012	Barriers and enablers to physical activity among older Australians who think they are insufficiently active	Macniven, R.; Pye, V.; Merom, D.; Milat, A.; Monger, C.; Bauman, A.; Van Der Ploeg, H.	10.1016/j.jsams.2012.11.110	03. Not CVD
Madsen 2013	'This is a forever project': supporting lifestyle changes in a regional Queensland community‐based cardiac rehabilitation program	Madsen, W.	10.1071/PY11137	04. CVD prevalence
Maneze 2018	Negotiating health and chronic illness in Filipino‐Australians: a qualitative study with implications for health promotion	Maneze, D.; Ramjan, L.; DiGiacomo, M.; Everett, B.; Davidson, P. M.; Salamonson, Y.	10.1080/13557858.2017.1294656	03. Not CVD
Marmot 2000	Social determinants of health: from observation to policy	Marmot, M.		03. Not CVD
Marsden 2010	A multidisciplinary group programme in rural settings for community‐dwelling chronic stroke survivors and their carers: a pilot randomized controlled trial	Marsden, D.; Quinn, R.; Pond, N.; Golledge, R.; Neilson, C.; White, J.; McElduff, P.; Pollack, M.	10.1177/0269215509344268	07. Intervention
McCluskey 2015	Compliance with Australian stroke guideline recommendations for outdoor mobility and transport training by post‐inpatient rehabilitation services: an observational cohort study	McCluskey, A.; Ada, L.; Kelly, P. J.; Middleton, S.; Goodall, S.; Grimshaw, J. M.; Logan, P.; Longworth, M.; Karageorge, A.	10.1186/s12913‐015‐0952‐7	02. Not social health
McIde 2017	Unpacking high self‐discharge rates for aboriginal cardiac patients	McIde, K.; Kelly, J.; Dowling, A.; Keech, W.; Brown, A.	10.1016/j.hlc.2017.06.683	04. CVD Prevalence
McKenna 2009	Comparison of time use, role participation and life satisfaction of older people after stroke with a sample without stroke	McKenna, K.; Liddle, J.; Brown, A.; Lee, K.; Gustafsson, L.	10.1111/j.1440‐1630.2007.00728.x	02. Not social health
McLachlan 2010	Equity of access to CVD risk management using electronic clinical decision support in the coronary care unit	McLachlan, A.; Wells, S.; Furness, S.; Jackson, R.; Kerr, A.	10.1016/j.ejcnurse.2010.01.007	02. Not social health
McLaughlin 2000	Travelling sales: an occupational hazard?	McLaughlin, M.; Holley, L.		03. Not CVD
Meyer 2013	Differentiating between trust and dependence of patients with coronary heart disease: furthering the sociology of trust	Meyer, S. B.; Ward, P. R.	10.1080/13698575.2013.776017	02. Not social health
Milligan 1997	Health‐related behaviours and psycho‐social characteristics of 18 y‐old Australians	Milligan, R. A.; Burke, V.; Beilin, L. J.; Richards, J.; Dunbar, D.; Spencer, M.; Balde, E.; Gracey, M. P.		03. Not CVD
Mitchell 1992	A cross‐cultural assessment of perceived health problems in the elderly	Mitchell, R. A.; Imperial, E.; Zhuo, D.; Lu, Y.; Watts, G.; Kelleher, P.; Brunker, P.; Gass, G.; Cue, R.; Cross, J.		06. Social health not assessed as a predictor of CVD
Mitchell 2014	The healthy neighbourhood audit instrument: understanding the environmental and socio‐cultural conditions to support healthy, happy and resilient residential communities	Mitchell, E.; Thompson, S.; Rowley, S.; Ong, R.; Markkanen, S.; Australian Government, Department of Families Housing Community Services; Indigenous, Affairs; Community Housing Coalition, W. A.; Government of Western Australia, Department of Housing; Shelter, W. A.; Stockland,		03. Not CVD
Moorley 2015	Life after stroke: releasing the cultural hostage	Moorley, C.	10.1111/ijs.12584	01. Not AUS/NZ
Morris 1991	The relationship between the perception of social support and post‐stroke depression in hospitalized patients	Morris, P. L. P.; Robinson, R. G.; Raphael, B.; Bishop, D.		04. CVD prevalence
Murphy 2013	Are poor health behaviours in anxious and depressed cardiac patients explained by sociodemographic factors?	Murphy, B. M.; Grande, M. R.; Navaratnam, H. S.; Higgins, R. O.; Elliott, P. C.; Turner, A.; Rogerson, M. C.; Worcester, M. U.; Goble, A. J.	10.1177/2047487312449593	04. CVD prevalence
Murphy 2014	Red flags for persistent or worsening anxiety and depression after an acute cardiac event: a 6‐mo longitudinal study in regional and rural Australia	Murphy, B.; Ludeman, D.; Elliott, P.; Judd, F.; Humphreys, J.; Edington, J.; Jackson, A.; Worcester, M.	10.1177/2047487313493058	04. CVD Prevalence
Myburgh 2018	Coping and cardiac troponin T: a risk for hypertension and sub‐clinical ECG left ventricular hypertrophy: the SABPA Study	Myburgh, C. E.; Malan, L.; Wentzel, A.; Scheepers, J. D. W.; Malan, N. T.	10.1016/j.hlc.2018.05.101	03. Not CVD
Nelson 2019	Developing cardiovascular risk prediction models for Australia	Nelson, M. R.; Woodward, M.	10.5694/mja2.50010	02. Not social health
O'Mara 2012	The spirit of the tent embassy: 40 y on indigenous self‐determination is essential to health and wellbeing	O'Mara, P.	10.5694/mja12.10829	03. Not CVD
Paisley 2008	Dietary change: what are the responses and roles of significant others?	Paisley, J.; Beanlands, H.; Goldman, J.; Evers, S.; Chappell, J.	10.1016/j.jneb.2007.04.374	03. Not CVD
Patel 2014	A multifaceted quality improvement intervention for CVD risk management in Australian primary healthcare: a protocol for a process evaluation	Patel, B.; Patel, A.; Jan, S.; Usherwood, T.; Harris, M.; Panaretto, K.; Zwar, N.; Redfern, J.; Jansen, J.; Doust, J.; Peiris, D.	10.1186/s13012‐014‐0187‐8	07. Intervention
Patel 2014	Understanding the impact of a multifaceted quality improvement intervention to improve cardiovascular disease risk management in Australian primary health care: the TORPEDO study process evaluation	Patel, B.; Usherwood, T.; Harris, M.; Panaretto, K.; Redfern, J.; Jansen, J.; McKinn, S.; Lyford, M.; Patel, A.; Peiris, D.	://dx.doi.org/10.1016/j.gheart.2014.03.2002	07. Intervention
Patel 2017	Impact of sustained use of a multifaceted computerized quality improvement intervention for cardiovascular disease management in Australian primary health care	Patel, B.; Peiris, D.; Usherwood, T.; Li, Q.; Harris, M.; Panaretto, K.; Zwar, N.; Patel, A.	10.1161/JAHA.117.007093	07. Intervention
Patel 2018	What drives adoption of a computerised, multifaceted quality improvement intervention for cardiovascular disease management in primary healthcare settings? A mixed methods analysis using normalisation process theory	Patel, B.; Usherwood, T.; Harris, M.; Patel, A.; Panaretto, K.; Zwar, N.; Peiris, D.	10.1186/s13012‐018‐0830‐x	02. Not social health
Patterson 2009	Long‐term stroke survivorsâ^™^ needs and perceptions of an exercise maintenance model of care	Patterson, S.; Ross‐Edwards, B.	10.12968/ijtr.2009.16.12.45422	04. CVD prevalence
Patterson 2010	Stroke maintenance exercise group: pilot study on daily functioning in long‐term stroke survivors	Patterson, S. A.; Ross‐Edwards, B. M.; Gill, H. L.	10.1071/PY09055 10.1046/j.1365‐2648.2000.01517.x	07. Intervention
Peiris 2009	An electronic clinical decision support tool to assist primary care providers in cardiovascular disease risk management: development and mixed methods evaluation	Peiris, D. P.; Joshi, R.; Webster, R. J.; Groenestein, P.; Usherwood, T. P.; Heeley, E.; Turnbull, F. M.; Lipman, A.; Patel, A. A.		02. Not social health
Peiris 2014	Effect of a multi‐faceted quality improvement intervention to improve cardiovascular disease risk identification and management in Australian Primary Health Care: the torpedo cluster‐randomised trial	Peiris, D.; Usherwood, T.; Panaretto, K.; Harris, M.; Hunt, J.; Zwar, N.; Redfern, J.; Cass, A.; Colagiuri, S.; Hayman, N.; Patel, A.	10.1016/j.gheart.2014.03.1317	07. Intervention
Peiris 2015	Effect of a computer‐guided, quality improvement program for cardiovascular disease risk management in primary health care: the treatment of cardiovascular risk using electronic decision support cluster‐randomized trial	Peiris, D.; Usherwood, T.; Panaretto, K.; Harris, M.; Hunt, J.; Redfern, J.; Zwar, N.; Colagiuri, S.; Hayman, N.; Lo, S.; Patel, B.; Lyford, M.; Macmahon, S.; Neal, B.; Sullivan, D.; Cass, A.; Jackson, R.; Patel, A.	10.1161/CIRCOUTCOMES.114.001235	07. Intervention
Penn 2017	Intercultural aphasia: new models of understanding for Indigenous populations	Penn, C.; Armstrong, E.	10.1080/02687038.2016.1213788	03. Not CVD
Petrie 1996	Role of patients' view of their illness in predicting return to work and functioning after myocardial infarction: longitudinal study	Petrie, K. J.; Weinman, J.; Sharpe, N.; Buckley, J.		04. CVD prevalence
Pettman 2008	Self‐management for obesity and cardio‐metabolic fitness: description and evaluation of the lifestyle modification program of a randomised controlled trial	Pettman, T. L.; Misan, G. M. H.; Owen, K.; Warren, K.; Coates, A. M.; Buckley, J. D.; Howe, P. R. C.	10.1186/1479‐5868‐5‐53	07. Intervention
Pier 2008	Identifying the health and mental health information needs of people with coronary heart disease, with and without depression	Pier, C.; Shandley, K. A.; Fisher, J. L.; Burstein, F.; Nelson, M. R.; Piterman, L.		04. CVD prevalence
Pit 2010	Health problems and retirement due to ill‐health among Australian retirees aged 45‐64 y	Pit, S. W.; Shrestha, R.; Schofield, D.; Passey, M.	10.1016/j.healthpol.2009.09.003	03. Not CVD
Pitama 2011	A Kaupapa Maori approach to a community cohort study of heart disease in New Zealand	Pitama, S.; Wells, J. E.; Faatoese, A.; Tikao‐Mason, K.; Robertson, P.; Huria, T.; Gillies, T.; Doughty, R.; Whalley, G.; Troughton, R.; Sheerin, I.; Richards, M.; Cameron, V. A.	10.1111/j.1753‐6405.2011.00702.x	02. Not social health
Provance 2019	Assessing patient preferences for shared decision‐making in peripheral artery disease: insights from the PORTRAIT Registry	Provance, J. B.; Spertus, J. A.; Decker, C.; Jones, P. G.; Smolderen, K. G.	10.1161/CIRCOUTCOMES.119.005730	01. Not AUS/NZ
Quigley 2019	Are we there yet? Exploring the journey to quality stroke care for Aboriginal and Torres Strait Islander peoples in rural and remote Queensland	Quigley, R.; Mann, J.; Robertson, J.; Bonython‐Ericson, S.	10.22605/RRH4850	04. CVD prevalence
Quirk 2018	Predictors of physical activity among rural adults following cardiac rehabilitation	Quirk, J.; Parfitt, G.; Ferrar, K.; Davison, K.; Dollman, J.	10.1037/rep0000232	04. CVD prevalence
Rabanal 2018	Performance of a Framingham cardiovascular risk model among Indians and Europeans in New Zealand and the role of body mass index and social deprivation	Rabanal, K. S.; Meyer, H. E.; Pylypchuk, R.; Mehta, S.; Selmer, R. M.; Jackson, R. T.	10.1136/openhrt‐2018‐000821	02. Not social health
Ramsamy 2017	A retrospective audit of post discharge outcome for patients supported by 'acute coronary syndrome support network' to remote communities in the northern territory of australia	Ramsamy, L.; Lau, S.; Abeyaratne, A.; Haste, M.; Kangaharan, N.	10.1016/j.hlc.2017.06.664	02. Not social health
Ray 2001	Self‐reported heart health behaviour patterns in a rural context	Ray, R.		03. Not CVD
Reilly 2008	Identifying psychosocial mediators of health amongst indigenous Australians for the Heart Health Project	Reilly, R. E.; Doyle, J.; Bretherton, D.; Rowley, K. G.; Harvey, J. L.; Briggs, P.; Charles, S.; Calleja, J.; Patten, R.; Atkinson, V.	10.1080/13557850801903046	04. CVD prevalence
Riddell 2012	Cluster randomized controlled trial of a peer support program for people with diabetes: study protocol for the Australasian peers for progress study	Riddell, M. A.; Renwick, C.; Wolfe, R.; Colgan, S.; Dunbar, J.; Hagger, V.; Absetz, P.; Oldenburg, B.	10.1186/1471‐2458‐12‐843	03. Not CVD
Riddell 2016	Cardiovascular risk outcome and program evaluation of a cluster randomised controlled trial of a community‐based, lay peer led program for people with diabetes	Riddell, M. A.; Dunbar, J. A.; Absetz, P.; Wolfe, R.; Li, H.; Brand, M.; Aziz, Z.; Oldenburg, B.; Oldenburg, B.; Dunbar, J. A.; Reddy, P.; Hagger, V.; Johnson, G.; De Courten, M.; Wolfe, R.; Carter, R.; Absetz, P.; Zaini, A.	10.1186/s12889‐016‐3538‐3	03. Not CVD
Rosbergen 2017	Embedding an enriched environment in an acute stroke unit increases activity in people with stroke: a controlled before‐after pilot study	Rosbergen, I. C. M.; Grimley, R. S.; Hayward, K. S.; Walker, K. C.; Rowley, D.; Campbell, A. M.; McGufficke, S.; Robertson, S. T.; Trinder, J.; Janssen, H.; Brauer, S. G.	10.1177/0269215517705181	07. Intervention
Rosbergen 2019	The impact of environmental enrichment in an acute stroke unit on how and when patients undertake activities	Rosbergen, I. C.; Grimley, R. S.; Hayward, K. S.; Brauer, S. G.	10.1177/0269215518820087	07. Intervention
Ryan 2017	A cross‐sectional study of work‐related and lifestyle factors associated with the health of Australian long distance commute and residential miners	Ryan, F.; Otto, B.; Khan, A.; Johnston, V.	10.1080/21679169.2017.1381324	03. Not CVD
Sapuppo 2018	The unmet needs of young stroke survivors	Sapuppo, D.; Thijs, V.; Bernhardt, J.	10.1177/1747493018778666	04. CVD prevalence
Schulz 2000	Factors which influence attendance at a rural Australian cardiac rehabilitation program	Schulz, D. L.; McBurney, H.	10.1054/chec.2000.0086	04. CVD prevalence
Scott 2004	Achieving better in‐hospital and after‐hospital care of patients with acute cardiac disease	Scott, I. A.; Denaro, C. P.; Bennett, C. J.; Hickey, A. C.; Mudge, A. M.; Flores, J. L.; Sanders, D. C. J.; Thiele, J. M.; Wenck, B.; Bennett, J. W.; Jones, M. A.		07. Intervention
Scott 2015	Body mass, cardiovascular risk and metabolic characteristics of young persons presenting for mental healthcare in Sydney, Australia	Scott, E. M.; Hermens, D. F.; White, D.; Naismith, S. L.; GeHue, J.; Whitwell, B. G.; Glozier, N.; Hickie, I. B.	10.1136/bmjopen‐2014‐007066	03. Not CVD
Simons 1998	Risk factors for ischemic stroke Dubbo study of the elderly	Simons Leon A. MD; John McCallum, DPhil; Yechiel Friedlander, PhD; Judith Simons, MACS		02. Not social health *identified through references
Simons 2013	Impact of loneliness and living alone	Simons, L. A.; McCallum, J.; Simons, J.		06. Social health not assessed as a predictor of CVD
Sinnett 1978	Lifestyle, health and disease: a comparison between Papua New Guinea and Australia	Sinnett, P.; Whyte, M.		03. Not CVD
Ski 2007	Stroke: the increasing complexity of carer needs	Ski, C.; O'Connell, B.		03. Not CVD
Son 2016	Biopsychosocial predictors of coping strategies of patients postmyocardial infarction	Son, H.; Friedmann, E.; Thomas, S. A.; Son, Y. J.	10.1111/ijn.12465	04. CVD Prevalence
Son 2019	How do patients develop self‐care behaviors to live well with heart failure?: a focus group interview study	Son, Y. J.; Lee, Y. M.; Kim, E. Y.	10.1016/j.colegn.2018.12.004	01. Not AUS/NZ
Spaderna 2017	Role of depression and social isolation at time of waitlisting for survival 8 y after heart transplantation	Spaderna, H.; Zittermann, A.; Reichenspurner, H.; Ziegler, C.; Smits, J.; Weidner, G.	10.1161/JAHA.117.007016	03. Not CVD
Spaeth 2018	Economic evaluation of point‐of‐care testing in the remote primary health care setting of Australia's Northern Territory	Spaeth, B. A.; Kaambwa, B.; Shephard, MD S.; Omond, R.	10.2147/CEOR.S160291	02. Not social health
Speechly 2010	Patient and general practitioner attitudes to healthy lifestyle behaviours and medication following coronary heart disease: An exploratory study	Speechly, C.; Bridges‐Webb, C.; McKenzie, S.; Zurynski, Y.; Lucas, A.	10.1071/PY09011	04. CVD prevalence
Stapelberg 2011	A topographical map of the causal network of mechanisms underlying the relationship between major depressive disorder and coronary heart disease	Stapelberg, N. J. C.; Neumann, D. L.; Shum, D. H. K.; McConnell, H.; Hamilton‐Craig, I.	://dx.doi.org/10.3109/00048674.2011.570427	04. CVD prevalence
Stewart 2003	Depression and cardiovascular morbidity and mortality: cause or consequence?	Stewart, R. A. H.; North, F. M.; West, T. M.; Sharples, K. J.; Simes, R. J.; Colquhoun, D. M.; White, H. D.; Tonkin, A. M.	10.1016/j.ehj.2003.08.017	04. CVD prevalence
Strodl 2013	A history of heart interventions moderates the relationship between psychological variables and the presence of chest pain in older women with self‐reported coronary heart disease	Strodl, E.; Kenardy, J.	10.1111/bjhp.12011	04. CVD prevalence
Stuart 2014	A telephone‐supported cardiovascular lifestyle programme (CLIP) for lipid reduction and weight loss in general practice patients: a randomised controlled pilot trial	Stuart, K. L.; Wyld, B.; Bastiaans, K.; Stocks, N.; Brinkworth, G.; Mohr, P.; Noakes, M.	://dx.doi.org/10.1017/S1368980013000220	07. Intervention
Tamplin 2013	'Stroke a chord': The effect of singing in a community choir on mood and social engagement for people living with aphasia following a stroke	Tamplin, J.; Baker, F. A.; Jones, B.; Way, A.; Lee, S.	://dx.doi.org/10.3233/NRE‐130916	03. Not CVD
Tavener 2015	Acknowledging how older australian women experience life after stroke: how does the WHO 18‐Item brief ICF core set for stroke compare?	Tavener, M.; Thijsen, A.; Hubbard, I. J.; Francis, J. L.; Grennall, C.; Levi, C.; Byles, J.	10.1080/07399332.2015.1055747	04. CVD prevalence
Taylor 2014	Implementing the evidence: from presumption to training	Taylor, E.; Chan, J.	10.1111/ijs.12334	04. CVD prevalence
Thompson 2017	Gender disparities in cardiovascular disease prevention	Thompson, L. E.; Daugherty, S. L.	10.1136/heartjnl‐2016‐310788	02. Not social health
Thurston 2008	What happens next? The role of cardiac rehabilitation in total patient care	Thurston, N.	10.1016/j.hlc.2008.09.005	07. Intervention
Tibby 2010	Establishment of an innovative specialist cardiac indigenous outreach service in rural and remote Queensland	Tibby, D.; Corpus, R.; Walters, D. L.	10.1016/j.hlc.2010.02.023	02. Not social health
Tideman 2014	Impact of a regionalised clinical cardiac support network on mortality among rural patients with myocardial infarction	Tideman, P. A.; Tirimacco, R.; Senior, D. P.; Setchell, J. J.; Huynh, L. T.; Tavella, R.; Aylward, P. E.; Chew, D. P.		02. Not social health
Tse 2017	Reduction in retained activity participation is associated with depressive symptoms 3 mo after mild stroke: an observational cohort study	Tse, T.; Douglas, J.; Lentin, P.; LindÃ©n, T.; Churilov, L.; Ma, H.; Davis, S.; Donnan, G.; Carey, L. M.	10.2340/16501977‐2184	04. CVD prevalence
Tse 2018	Longitudinal changes in activity participation in the first year post‐stroke and association with depressive symptoms	Tse, T.; Linden, T.; Churilov, L.; Davis, S.; Donnan, G.; Carey, L. M.	10.1080/09638288.2018.1471742	04. CVD prevalence
Tse 2019	Longitudinal changes in activity participation in the first year post‐stroke and association with depressive symptoms	Tse, T.; Linden, T.; Churilov, L.; Davis, S.; Donnan, G.; Carey, L. M.	10.1080/09638288.2018.1471742	04. CVD prevalence
Tully 2014	Routine depression screening after cardiac surgery simply misses those that need it most: Impact of missed cases on hospital resource utilization, cardiac outcomes, depression and quality of life	Tully, P.; Baker, R. A.	10.1177/1474515114521363	04. CVD prevalence
Turner 2010	Clinical outcomes associated with depression, anxiety and social support among cardiac rehabilitation attendees	Turner, A.; Phillips, L.; Hambridge, J. A.; Baker, A. L.; Bowman, J.; Colyvas, K.	10.3109/00048671003646751	04. CVD prevalence
Unsworth 1995	Rehabilitation team decisions on discharge housing for stroke patients	Unsworth, C. A.; Thomas, S. A.; Greenwood, K. M.	10.1016/S0003‐9993(95)80658‐X	04. CVD prevalence
Unsworth 1996	Clients' perceptions of discharge housing decisions after stroke rehabilitation	Unsworth, C.	10.5014/ajot.50.3.207	04. CVD prevalence
Unsworth 2019	Preliminary screening recommendations for patients at risk of depression and/or anxiety more than 1 y poststroke	Unsworth, David J.; Mathias, Jane L.; Dorstyn, Diana S.	10.1016/j.jstrokecerebrovasdis.2019.03.014	04. CVD prevalence
Vallesi 2018	"In their own voice"‐incorporating underlying social determinants into aboriginal health promotion programs	Vallesi, S.; Wood, L.; Dimer, L.; Zada, M.	10.3390/ijerph15071514	03. Not CVD
VonDohren 2013	Taking therapeutic leisure and recreation seriously during stroke recovery	Von Dohren, C.; Chin, G.	10.1111/ijs.12172	06. Social health not assessed as a predictor of CVD
Wang 2010	The prevalence and predictors of anxiety and depression in adolescents with heart disease	Wang, Q.; Hay, M.; Clarke, D.; Menahem, S.	10.1111/j.1445‐5994.2010.02186.x	04. CVD prevalence
Wang 2011	Psychosocial functioning in adolescents with heart disease	Wang, Q.; Hay, M.; Clarke, D.; Menahem, S.	10.1016/j.hlc.2011.05.599	04. CVD prevalence
Ward 2011	With good intentions: complexity in unsolicited informal support for Aboriginal and Torres Strait Islander peoples. A qualitative study	Ward, N. J.; Jowsey, T.; Haora, P. J.; Aspin, C.; Yen, L. E.	10.1186/1471‐2458‐11‐686	03. Not CVD
Wells 2017	Cohort profile: the PREDICT cardiovascular disease cohort in New Zealand primary care (PREDICT‐CVD 19)	Wells, S.; Riddell, T.; Kerr, A.; Pylypchuk, R.; Chelimo, C.; Marshall, R.; Exeter, D. J.; Mehta, S.; Harrison, J.; Kyle, C.; Grey, C.; Metcalf, P.; Warren, J.; Kenealy, T.; Drury, P. L.; Harwood, M.; Bramley, D.; Gala, G.; Jackson, R.	10.1093/ije/dyv312	02. Not social health
Westbrook 1993	Attitudes towards disabilities in a multicultural society	Westbrook, M. T.; Legge, V.; Pennay, M.		03. Not CVD
Wheeler 2012	Depression and 5‐y mortality in patients with acute myocardial infarction: analysis of the IDACC database	Wheeler, A.; Beltrame, J.; Tucker, G.; Air, T.; Ling, L. H.; Schrader, G.	10.1177/0004867412449875	04. CVD prevalence
White 2007	Community‐dwelling stroke survivors: function is not the whole story with quality of life	White, J. H.; Alston, M. K.; Marquez, J. L.; Sweetapple, A. L.; Pollack, M. R.; Attia, J.; Levi, C. R.; Sturm, J.; Whyte, S.		04. CVD prevalence
White 2008	The occupational experience of stroke survivors in a community setting	White, J. H.; MacKenzie, L.; Magin, P.; Pollack, M. R. P.	10.3928/15394492‐20080901‐05	04. CVD prevalence
White 2009	Exploring poststroke mood changes in community‐dwelling stroke survivors: a prospective, longitudinal, mixed methods study	White, J.; Magin, P.; Attia, J.; Sturm, J.; Pollack, M.; McElduff, P.	10.1111/j.1747‐4949.2009.00306.x	04. CVD prevalence
White 2016	Predictors of health‐related quality of life in community‐dwelling stroke survivors: a cohort study	White, J.; Magin, P.; Attia, J.; Sturm, J.; McElduff, P.; Carter, G.	10.1093/fampra/cmw011	06. Social health not assessed as a predictor of CVD
Wilson 1993	The good heart, good life survey: self‐reported cardiovascular disease risk factors, health knowledge and attitudes among Greek‐Australians in Sydney	Wilson, A.; Bekiaris, J.; Gleeson, S.; Papasavva, C.; Wise, M.; Hawe, P.	10.1111/j.1753‐6405.1993.tb00138.x	03. Not CVD
Winefield 1982	Male social support and recovery after myocardial infarction	Winefield, H. R.	10.1080/00049538208254716	04. CVD prevalence
Wong 2010	Caveat anicula! Beware of quiet little old ladies: demographic features, pharmacotherapy, readmissions and survival in a 10‐y cohort of patients with heart failure and preserved systolic function	Wong, D. T.; Clark, R. A.; Dundon, B. K.; Philpott, A.; Molaee, P.; Shakib, S.		04. CVD prevalence
Worrall‐Carter 2005	The experiences and adjustments of women following their first acute myocardial infarction	Worrall‐Carter, L.; Jones, T.; Driscoll, A.		04. CVD prevalence
Yarmo‐Roberts 2010	The heart of the matter: health status of aged care clients receiving home‐ and community‐based care	Yarmo‐Roberts, D.; Freak‐Poli, R. L.; Cooper, B.; Noonan, T.; Stolewinder, J.; Reid, C. M.	10.4061/2010/275303	03. Not CVD
Zecchin 2016	Cardiac rehabilitation for patients with spontaneous coronary artery dissection	Zecchin, R.; Thelander, J.; Baihn, J.; Chai, Y.; Haeusler, K.; Hungerford, J.; Lindsay, G.; Pettitt, M.; Vail, T.; Cooper, M.; Ong, A.; Chow, C.; Denniss, R.	10.1016/j.hlc.2016.06.768	04. CVD prevalence
Zhang 2017	Using the ‘Think Aloud’ technique to explore quality of life issues during standard quality‐of‐life questionnaires in patients with atrial fibrillation	Zhang, L.; Gallagher, R.; Lowres, N.; Orchard, J.; Freedman, S. B.; Neubeck, L.	10.1016/j.hlc.2016.05.121	07. Intervention

## APPENDIX C

### Risk of bias

**Table:**

Authors	Selection				Comparability		Outcome			Overall	
	1	2	3	4	1		1	2	3		(9 scores)
Strodl 2003^27^	a*	a*	b*	a*		b*	c	b	b*		6
Strodl 2008^28^	a*	a*	b*	a*		b*	c	b	b*		6
Byles 2015^39^	a*	a*	b*	b			c	b	a*		4
Simons 2013^40^	a*	a*	b*	b		b*	b*	a*	a*		7
Sahle 2020^45^	a*	a*	b*	a*		b*	c	a*	b*		7

Strodl 2008^28^ was provided a point for Comparability “*control for any additional factors*” as multivariable adjustment would have been undertaken if social health was statistically significant in univariable analyses.

Appraisal Standard of Newcastle/Ottawa Scale (NOS). * indicates 1 point. The NOS includes eight items, categorised into three dimension (Selection, Comparability, and Outcome) and provides a rating from 0 to 9 stars, with higher scores indicating less susceptibility to bias. To undertake the NOS, components of two items had to be further clarified or specified. To assess “*Comparability of cohorts on the basis of the design or analysis*” we defined *“Study control for most important factors*” as age, sex, sociodemographic (eg education or SEIFA), smoking, blood pressure, cholesterol, and diabetes. We defined “*Study control for any additional factors*” as either ethnicity, remoteness, lifestyle (alcohol, diet, physical activity), partner status, or mental health. To assess “*Was follow‐up long enough for outcomes to occur*” we defined “*Yes*” as 5 y or more of follow‐up.

**Table:**

Content
Selection
**Representativeness of the exposed cohort** Truly representative of the average general population with or without health conditions * Somewhat representative of the average general population with or without health conditions * Selected group of users No description of the derivation of the cohort
**Selection of the non‐exposed group/cohort** Drawn from the same community as the exposed cohort * Drawn from a different source No description of the derivation of the non‐exposed group
**Ascertainment of Social Health exposure** Secure record * (not possible) Structured interview or questionnaire * Written self‐reports No description
**Demonstration that Cardiovascular disease outcome of interest was not present at the start of study** Yes * No
Comparability (2 stars possible)
**Comparability of cohorts on the basis of the design or analysis** Study control for most important factors – * defined as age, sex, sociodemographic (eg education or SEIFA), smoking, blood pressure, cholesterol, **and** diabetes Study control for any additional factors – * Either: ethnicity, remoteness, lifestyle (alcohol, diet, physical activity), partner status, **or** mental health
Cardiovascular disease outcome
**Assessment of cardiovascular disease outcome** Independent blind assessment * Not possible Record linkage * Self‐report No description
**Was follow‐up long enough for outcomes to occur** Yes (More than and equal to 5‐y follow‐up) * No
**Adequacy of follow‐up of cohorts** Complete follow up – all subjects accounted for * Subjects lost to follow‐up unlikely to introduce bias – small number lost ‐ >80% follow up, or description provided for those lost * Follow‐up <80% and no description of those lost No statement

## APPENDIX D

### Funnel plot of social health as a predictor of cardiovascular disease, conversion to relative risk

The Egger's test demonstrated that there was no small‐study effects (regress standard normal deviate of intervention effect estimate against its standard error: slope 0.00 + 0.41SE, bias 0.44 + 3.85SE, *P* = .919).

## References

[1] Australian Institute of Health and Welfare . Cardiovascular disease snapshot: Australian Institute of Health and Welfare Australian Government. 2018 [cited 2019 Mar 24]. Available from: https://www.aihw.gov.au/reports/heart‐stroke‐vascular‐disease/cardiovascular‐health‐compendium/contents/how‐many‐australians‐have‐cardiovascular‐disease

[2] The Ministry of Health . New Zealand Health Survey: Annual Data Explorer: November 2019. 2020 [cited 2020 Sep 3]. Available from: https://minhealthnz.shinyapps.io/nz‐health‐survey‐2018‐19‐annual‐data‐explorer

[3] Blakely T , Kvizhinadze G , Atkinson J , Dieleman J , Clarke P . Health system costs for individual and comorbid noncommunicable diseases: an analysis of publicly funded health events from New Zealand. PLoS Med. 2019;16(1):e1002716. 30620729 10.1371/journal.pmed.1002716 PMC6324792

[4] Bunker SJ , Colquhoun DM , Esler MD , Hickie IB , Hunt D , Jelinek VM , et al. "Stress" and coronary heart disease: psychosocial risk factors. Med J Aust. 2003;178(6):272–6. 12633484 10.5694/j.1326-5377.2003.tb05193.x

[5] National Heart Foundation of Australia . Strategy Plan 2013‐2017. 2012 [cited 2017 March 30]. Available from: https://heartfoundation.org.au/images/uploads/main/About_us/Heart‐Foundation‐Strategic‐Plan‐2013‐2017.pdf

[6] Freak‐Poli R , Ryan J , Tran T , Owen A , McHugh Power J , Berk M , et al. Social isolation, social support and loneliness as independent concepts, and their relationship with health‐related quality of life among older women. Aging Ment Health. 2021;1–10. 10.1080/13607863.2021.1940097 34219569

[7] National Academies of Sciences, Engineering, and Medicine . Social Isolation and Loneliness in Older Adults: Opportunities for the Health Care System. Washington, DC: The National Academies Press; 2020. 32510896

[8] Commissioner for Senior Victorians . Ageing is everyone’s business: a report on isolation and loneliness among senior Victorians. Melbourne: State of Victoria, Department of Health and Human Services; 2016.

[9] Courtin E , Knapp M . Social isolation, loneliness and health in old age: a scoping review. Health Soc Care Community. 2017;25(3):799–812. 26712585 10.1111/hsc.12311

[10] Valtorta NK , Kanaan M , Gilbody S , Hanratty B . Loneliness, social isolation and social relationships: what are we measuring? A novel framework for classifying and comparing tools. BMJ Open. 2016;6(4):e010799. 10.1136/bmjopen-2015-010799 PMC4838704 27091822

[11] Australian Institute of Health and Welfare (AIHW) . Australia’s welfare 2019 in brief. Cat. no. AUS 227. Canberra: AIHW; 2019.

[12] Ministry of Social Development . The Social Report 2016: Te pūrongo oranga tangata. Wellington: Ministry of Social Development; 2016 [cited 2020 Sep 3] Available from: http://socialreport.msd.govt.nz/documents/2016/msd‐the‐social‐report‐2016.pdf

[13] Hodgson S , Watts I , Fraser S , Roderick P , Dambha‐Miller H . Loneliness, social isolation, cardiovascular disease and mortality: a synthesis of the literature and conceptual framework. J R Soc Med. 2020;113(5):185–92. 32407646 10.1177/0141076820918236 PMC7366328

[14] Howick J , Kelly P , Kelly M . Establishing a causal link between social relationships and health using the Bradford Hill Guidelines. SSM Popul Health. 2019;8:100402. 31193417 10.1016/j.ssmph.2019.100402 PMC6527915

[15] Holt‐Lunstad J , Smith TB . Loneliness and social isolation as risk factors for CVD: implications for evidence‐based patient care and scientific inquiry. Heart. 2016;102(13):987–9. 27091845 10.1136/heartjnl-2015-309242 PMC4941164

[16] Berkman LF , Glass T , Brissette I , Seeman TE . From social integration to health: Durkheim in the new millennium. Soc Sci Med. 2000;51(6):843–57. 10972429 10.1016/s0277-9536(00)00065-4

[17] Lim MH , Eres R , Vasan S . Understanding loneliness in the twenty‐first century: an update on correlates, risk factors, and potential solutions. Soc Psychiatry Psychiatr Epidemiol. 2020;55(7):793–810. 32524169 10.1007/s00127-020-01889-7

[18] Xia N , Li H . Loneliness, social isolation, and cardiovascular health. Antioxid Redox Signal. 2018;28(9):837–51. 28903579 10.1089/ars.2017.7312 PMC5831910

[19] Ong AD , Uchino BN , Wethington E . Loneliness and health in older adults: a mini‐review and synthesis. Gerontology. 2016;62(4):443–9. 26539997 10.1159/000441651 PMC6162046

[20] Sharma T , Padala PR , Mehta JL . Loneliness and social isolation: determinants of cardiovascular outcomes. Curr Cardiol Rev. 2021;17(6):7–14. 10.2174/1573403X17666210129101845 PMC8950500 33511946

[21] Singh RA , Javed Z , Yahya T , Valero‐Elizondo J , Acquah I , Hyder AA , et al. Community and social context: an important social determinant of cardiovascular disease. Methodist DeBakey Cardiovasc J. 2021;17(4):15–27. 34824678 10.14797/mdcvj.846 PMC8588761

[22] Freak‐Poli R , Ryan J , Neumann JT , Tonkin A , Reid CM , Woods RL , et al. Social isolation, social support and loneliness as predictors of cardiovascular disease incidence and mortality. BMC Geriatr. 2021;21(1):711. 34922471 10.1186/s12877-021-02602-2 PMC8684069

[23] Williams LJ , Quirk SE , Koivumaa‐Honkanen H , Honkanen R , Pasco JA , Stuart AL , et al. Personality disorder and physical health comorbidities: a link with bone health? Front Psychiatry. 2020;11:602342. 33363487 10.3389/fpsyt.2020.602342 PMC7752862

[24] Cacioppo S , Capitanio JP , Cacioppo JT . Toward a neurology of loneliness. Psychol Bull. 2014;140(6):1464–504. 25222636 10.1037/a0037618 PMC5130107

[25] Hu J , Fitzgerald SM , Owen AJ , Ryan J , Joyce J , Chowdhury E , et al. Social isolation, social support, loneliness and cardiovascular disease risk factors: a cross‐sectional study among older adults. Int J Geriatr Psychiatry. 2021;36(11):1795–809. 34231940 10.1002/gps.5601

[26] Valtorta NK , Kanaan M , Gilbody S , Ronzi S , Hanratty B . Loneliness and social isolation as risk factors for coronary heart disease and stroke: systematic review and meta‐analysis of longitudinal observational studies. Heart. 2016;102(13):1009–16. 27091846 10.1136/heartjnl-2015-308790 PMC4941172

[27] Strodl E , Kenardy J , Aroney C . Perceived stress as a predictor of the self‐reported new diagnosis of symptomatic CHD in older women. Int J Behav Med. 2003;10(3):205–20. 14525717 10.1207/s15327558ijbm1003_02

[28] Strodl E , Kenardy J . The 5‐item mental health index predicts the initial diagnosis of nonfatal stroke in older women. J Womens Health. 2008;17(6):979–86. 10.1089/jwh.2007.0516 18681818

[29] Liberati A , Altman DG , Tetzlaff J , Mulrow C , Gotzsche PC , Ioannidis JP , et al. The PRISMA statement for reporting systematic reviews and meta‐analyses of studies that evaluate health care interventions: explanation and elaboration. J Clin Epidemiol. 2009;62(10):e1–e34. 19631507 10.1016/j.jclinepi.2009.06.006

[30] Strodl E . Personal communication with RF. 16 Jul 2020.

[31] Byles J . Personal communication with RF. 16 to 17 Jul 2020.

[32] Simons L . Personal communication with RF. 16 Jul to 2 Sep 2020.

[33] The Ottawa Hospital Research Institute . The Newcastle‐Ottawa Scale (NOS) for assessing the quality if nonrandomized studies in meta‐analyses. 2019 [cited 2020 Sep 3]. Available from: http://wwwohrica/programs/clinical_epidemiology/oxfordasp

[34] von Elm E , Altman DG , Egger M , Pocock SJ , Gøtzsche PC , Vandenbroucke JP ,; et al. The Strengthening the Reporting of Observational Studies in Epidemiology (STROBE) statement: guidelines for reporting observational studies. J Clin Epidemiol. 2008. 10.1016/j.jclinepi.2007.11.008 (0895‐4356 (Print)).

[35] Deeks JJ , Higgins JPT , Altman DG , editors. Chapter 10: Analysing data and undertaking meta‐analyses. In: Higgins JPT , Thomas J , Chandler J , Cumpston M , Li T , Page MJ , Welch VA , editors. Cochrane handbook for systematic reviews of interventions version 6.0 (updated July 2019). Cochrane. 2019 [cited 2020 Sep 1]. Available from: www.training.cochrane.org/handbook

[36] Schünemann HJ , Vist GE , Higgins JPT , Santesso N , Deeks JJ , Glasziou P , et al. Chapter 15: Interpreting results and drawing conclusions. In: Higgins JPT , Thomas J , Chandler J , Cumpston M , Li T , Page MJ , Welch VA , editors. Cochrane handbook for systematic reviews of interventions version 6.0 (updated July 2019). Cochrane, 2019. Available from: www.training.cochrane.org/handbook

[37] Shor E , Roelfs D , Vang ZM . The "Hispanic mortality paradox" revisited: meta‐analysis and meta‐regression of life‐course differentials in Latin American and Caribbean immigrants' mortality. Soc Sci Med. 2017;186:20–33. 28577458 10.1016/j.socscimed.2017.05.049

[38] Greenland S , Longnecker MP . Methods for trend estimation from summarized dose‐response data, with applications to meta‐analysis. Am J Epidemiol. 1992;135(11):1301–9. 1626547 10.1093/oxfordjournals.aje.a116237

[39] Byles JE , Francis JL , Chojenta CL , Hubbard IJ . Long‐term survival of older australian women with a history of stroke. J Stroke Cerebrovasc Dis. 2015;24(1):53–60. 25440353 10.1016/j.jstrokecerebrovasdis.2014.07.040

[40] Simons LA , McCallum J , Simons J . Impact of loneliness and living alone. JAMA Intern Med. 2013;173(4):322. 10.1001/jamainternmed.2013.1611 23440234

[41] Simons LA , Simons J , Friedlander Y , McCallum J . Predictors of long‐term mortality in the elderly: the Dubbo Study. Intern Med J. 2011;41(7):555–60. 19849748 10.1111/j.1445-5994.2009.02106.x

[42] Simons LA , McCallum J , Friedlander Y , Simons J , Powell I , Heller R . Dubbo study of the elderly: sociological and cardiovascular risk factors at entry. Aust N Z J Med. 1991;21(5):701–9. 1759918 10.1111/j.1445-5994.1991.tb01373.x

[43] Simons LA , McCallum J , Simons J , Powell I , Ruys J , Heller R , et al. The Dubbo study: an Australian prospective community study of the health of elderly. Aust N Z J Med. 1990;20(6):783–9. 2291727 10.1111/j.1445-5994.1990.tb00423.x

[44] Simons LA , Simons J , Friedlander Y , McCallum J . A comparison of risk factors for coronary heart disease and ischaemic stroke: the Dubbo study of Australian elderly. Heart Lung Circ. 2009;18(5):330–3. 19648057 10.1016/j.hlc.2009.05.001

[45] Sahle BW , Chen W , Melaku YA , Akombi BJ , Rawal LB , Renzaho AMN . Association of psychosocial factors with risk of chronic diseases: a nationwide longitudinal study. Am J Prev Med. 2020;58(2):e39–50. 31959325 10.1016/j.amepre.2019.09.007

[46] Caspi A , Harrington H , Moffitt TE , Milne BJ , Poulton R . Socially isolated children 20 years later ‐ risk of cardiovascular disease. Arch Pediatr Adolesc Med. 2006;160(8):805–11. 16894079 10.1001/archpedi.160.8.805

[47] Danese A , Moffitt TE , Harrington H , Milne BJ , Polanczyk G , Pariante CM , et al. Adverse childhood experiences and adult risk factors for age‐related disease depression, inflammation, and clustering of metabolic risk markers. Arch Pediatr Adolesc Med. 2009;163(12):1135–43. 19996051 10.1001/archpediatrics.2009.214 PMC3560401

[48] Australian Institute of Health and Welfare . Australia’s welfare 2017. Canberra: AIHW; 2017 [cited 2019 Jan 24]. Available from: www.AIHW.gov.au/getmedia/088848dc‐906d‐4a8b‐aa09‐79df0f943984/aihw‐aus‐214‐aw17.pdf.aspx?inline=true

[49] Havens B , Hall M , Sylvestre G , Jivan T . Social isolation and loneliness: differences between older rural and urban Manitobans. Can J Aging. 2004;23(2):129–40. 15334813 10.1353/cja.2004.0022

[50] Bolton M . Loneliness – the state we’re in: A report of evidence compiled for the Campaign to End Loneliness. 2012. [cited 2020 Oct 27]. Available from: www.campaigntoendloneliness.org/wp‐content/uploads/Loneliness‐The‐State‐Were‐In.pdf

[51] Department of Health and Ageing . Suicide in rural and remote communities. Fact Sheet 18. ACT: Australian Government; 2007. [cited 2019 Jan 24[. Available from: www.cranbrook.wa.gov.au/library/file/download%20documents/Suicide‐in‐rural‐and‐remote‐communities.pdf

[52] Beer A , Faulkner D , Law J , Lewin G , Tinker A , Buys L , et al. Regional variation in social isolation amongst older Australians. Reg Stud Reg Sci. 2016;3(1):170–84.

[53] Pretty G , Bishop B , Fisher A , Sonn C . Psychological sense of community and its relevance to well‐being and everyday life in Australia. Melbourne, Australia: The Australian Psychological Society Ltd.; 2006 [cited 2019 Feb 20]. Available from: http://groups.psychology.org.au/assets/files/community‐updated‐sept061.pdf

[54] Nicholls SJ , Nelson M , Astley C , Briffa T , Brown A , Clark R , et al. Optimising secondary prevention and cardiac rehabilitation for atherosclerotic cardiovascular disease during the COVID‐19 pandemic: a position Statement from the Cardiac Society of Australia and New Zealand (CSANZ). Heart Lung Circ. 2020;29(7):e99–e104. 32473781 10.1016/j.hlc.2020.04.007 PMC7192068

